# A Genome-Scale RNA–Interference Screen Identifies RRAS Signaling as a Pathologic Feature of Huntington's Disease

**DOI:** 10.1371/journal.pgen.1003042

**Published:** 2012-11-29

**Authors:** John P. Miller, Bridget E. Yates, Ismael Al-Ramahi, Ari E. Berman, Mario Sanhueza, Eugene Kim, Maria de Haro, Francesco DeGiacomo, Cameron Torcassi, Jennifer Holcomb, Juliette Gafni, Sean D. Mooney, Juan Botas, Lisa M. Ellerby, Robert E. Hughes

**Affiliations:** 1 The Buck Institute for Research on Aging, Novato, California, United States of America; 2 Department of Molecular and Human Genetics, Baylor College of Medicine, Houston, Texas, United States of America; Stanford University School of Medicine, United States of America

## Abstract

A genome-scale RNAi screen was performed in a mammalian cell-based assay to identify modifiers of mutant huntingtin toxicity. Ontology analysis of suppressor data identified processes previously implicated in Huntington's disease, including proteolysis, glutamate excitotoxicity, and mitochondrial dysfunction. In addition to established mechanisms, the screen identified multiple components of the RRAS signaling pathway as loss-of-function suppressors of mutant huntingtin toxicity in human and mouse cell models. Loss-of-function in orthologous RRAS pathway members also suppressed motor dysfunction in a Drosophila model of Huntington's disease. Abnormal activation of RRAS and a down-stream effector, RAF1, was observed in cellular models and a mouse model of Huntington's disease. We also observe co-localization of RRAS and mutant huntingtin in cells and in mouse striatum, suggesting that activation of R-Ras may occur through protein interaction. These data indicate that mutant huntingtin exerts a pathogenic effect on this pathway that can be corrected at multiple intervention points including RRAS, FNTA/B, PIN1, and PLK1. Consistent with these results, chemical inhibition of farnesyltransferase can also suppress mutant huntingtin toxicity. These data suggest that pharmacological inhibition of RRAS signaling may confer therapeutic benefit in Huntington's disease.

## Author Summary

Huntington's disease (HD) is an inherited disorder caused by mutation of the gene that encodes the huntingtin protein. The specific mutation that results in disease is an increase in the copies of the amino acid glutamine in a stretch of repeated glutamines at the amino-terminus of the protein. This “expanded polyglutamine” huntingtin acquires toxic properties, presumably through mechanisms that involve its reduced solubility and aberrant interactions with other cellular proteins that do not occur with the normal protein. In this study, we sought to identify cellular processes that were involved in the toxicity conferred by the mutant huntingtin protein. We used RNA interference in order to specifically reduce the levels of individual cellular proteins and identified a number that could reduce mutant huntingtin toxicity. These modifiers clustered into functional pathways know to be involved in HD and other novel pathways. Among these modifiers, we found that the signaling protein RRAS, as well as additional members of its signaling cascade, are involved in mutant huntingtin toxicity. We further showed that a small molecule inhibitor of an enzyme involved in this pathway is effective at reducing this toxicity, indicating that the targeted inhibition of the RRAS pathway may be of therapeutic benefit in Huntington's disease.

## Introduction

Huntington's disease (HD) is a dominantly-inherited, invariably fatal, familial neurodegenerative disease caused by an expansion in the polyglutamine encoding CAG tract in the huntingtin gene (Htt) [1]. HD manifests with severe motor and psychiatric impairments caused by neuronal dysfunction and loss in the cortex and striatum [2]. Mutant Htt causes cellular dysfunction through mechanisms involving a toxic gain-of-function of the mutant protein. However, loss of neural-protective functions provided by the wild-type protein may also contribute to the disease phenotype [3]. Pathways and processes disrupted by mutant Htt include transcription [4], mitochondrial bioenergetics and metabolism [5], and proteasomal degradation [6]. Additionally, signaling cascades that have yet to be implicated may impinge on multiple defective processes in HD. There is currently no therapeutic treatment for HD, and a significant challenge is the identification of cellular drug targets for this disease.

In order to comprehensively discover novel drug targets for HD, we completed a large-scale RNAi screen in a human cell-based model of mutant huntingtin toxicity. Similar approaches have been used to map modifier pathways in cancer, and infectious disease models [7], [8]. Modifiers identified in this screen were systematically validated in higher content models including a mouse *Hdh* knock-in cell model [9] of cell death, and a *Drosophila* model of HD motor dysfunction [10]. The primary screen identified a number of pathways and biological processes known to be involved in HD, indicating that the cell-model and modifier results are generally relevant to molecular aspects of the disease. Subsequent validation of novel targets demonstrate that augmented signaling though RRAS and downstream effectors, may be a druggable pathological feature of HD.

## Results

### A Genome-Scale siRNA Screen for Suppressors of Mutant Htt Toxicity

To discover proteins and pathways that modify mutant Htt toxicity, we carried out a siRNA screen in cells expressing the N-terminal 558 amino acids of mutant Htt fused to GFP (Htt_1-558_141Q-GFP). HEK293T cells expressing this mutant Htt fragment show rounding and detachment indicative of toxicity (data not shown), and enhanced caspase activation upon growth factor deprivation relative to control cells (Figure S1). To perform the screen, we co-transfected the Htt_1-558_141Q-GFP construct with 7,494 unique siRNA pools, each targeting the product of a gene identified as pharmacologically tractable by empirical and/or homology-based analyses (the Dharmacon Druggable Genome Set), as well as overlapping sets of kinase, G-protein coupled receptor (GPCR), and protease gene families. The effect of each siRNA pool on caspase activation in response to serum-withdrawal was measured, and pools showing significant suppression of caspase activation. This was measured by caspase 3/7 activity, and control wells transfected with siRNA against *CASP3* served as a positive control (Figure S1). The results for the entire screen are presented in Table S1. The top 130 siRNA hits from the screen that caused a reduction of more than 1 standard deviation below the mean for the entire screen are shown in Table 1. These are ranked according to the average magnitude of suppression of caspase activity.

**Table 1:** List of Suppressors.

siRNA	GENEID	Entrez Gene Name	Function	AVG^a^	SE^b^
IL18BP	10068	interleukin 18 binding protein	other	0.187	0.001
GRIN1	2902	glutamate receptor, ionotropic, N-methyl D-aspartate 1	ion channel	0.213	0.041
NR3C2	4306	nuclear receptor subfamily 3, group C, member 2	nuclear receptor	0.258	0.037
TNFSF18	8995	tumor necrosis factor (ligand) superfamily, member 18	cytokine	0.275	0.032
TNFSF13B	10673	tumor necrosis factor (ligand) superfamily, member 13b	cytokine	0.298	0.094
ENPEP	2028	glutamyl aminopeptidase (aminopeptidase A)	peptidase	0.299	0.036
MIPEP	4285	mitochondrial intermediate peptidase	peptidase	0.320	0.059
GDF10	2662	growth differentiation factor 10	growth factor	0.328	0.023
GAS	2520	gastrin	other	0.329	0.050
CPA1	1357	carboxypeptidase A1 (pancreatic)	peptidase	0.333	0.038
HRH2	3274	histamine receptor H2	GPCR	0.376	0.060
HRH1	3269	histamine receptor H1	GPCR	0.377	0.156
UCHL3	7347	ubiquitin carboxyl-terminal esterase L3 (ubiquitin thiolesterase)	peptidase	0.393	0.013
CASP3	836	caspase 3, apoptosis-related cysteine peptidase	peptidase	0.405	0.017
P2RX4	5025	purinergic receptor P2X, ligand-gated ion channel, 4	ion channel	0.420	0.081
LTBR	4055	lymphotoxin beta receptor (TNFR superfamily, member 3)	transmembrane receptor	0.422	0.027
LGALS9	3965	lectin, galactoside-binding, soluble, 9	other	0.425	0.158
DPEP3	64180	dipeptidase 3	peptidase	0.451	0.045
ADAM9	8754	ADAM metallopeptidase domain 9 (meltrin gamma)	peptidase	0.451	0.015
IL18R1	8809	interleukin 18 receptor 1	transmembrane receptor	0.463	0.016
GPRC5D	55507	G protein-coupled receptor, family C, group 5, member D	GPCR	0.465	0.120
IL1RAP	3556	interleukin 1 receptor accessory protein	transmembrane receptor	0.478	0.113
USP1	7398	ubiquitin specific peptidase 1	peptidase	0.483	0.016
NR2F6	2063	nuclear receptor subfamily 2, group F, member 6	nuclear receptor	0.487	0.051
NAGLU	4669	N-acetylglucosaminidase, alpha	enzyme	0.487	0.148
DLX4	1748	distal-less homeobox 4	transcription regulator	0.489	0.128
MSLN	10232	mesothelin	other	0.489	0.052
AP1GBP1	11276	synergin, gamma	other	0.492	0.135
OR5P2	120065	olfactory receptor, family 5, subfamily P, member 2	GPCR	0.493	0.057
LHCGR	3973	luteinizing hormone/choriogonadotropin receptor	GPCR	0.499	0.166
PSMB5	5693	proteasome (prosome, macropain) subunit, beta type, 5	peptidase	0.503	0.087
MAP3K14	9020	mitogen-activated protein kinase kinase kinase 14	kinase	0.507	0.012
LHB	3972	luteinizing hormone beta polypeptide	other	0.507	0.040
PDE9A	5152	phosphodiesterase 9A	enzyme	0.511	0.121
PPP1R3D	5509	protein phosphatase 1, regulatory (inhibitor) subunit 3D	phosphatase	0.518	0.014
GLRA2	2742	glycine receptor, alpha 2	ion channel	0.520	0.101
MLL5	55904	myeloid/lymphoid or mixed-lineage leukemia 5 (trithorax homolog, Drosophila)	other	0.521	0.051
NR3C1	2908	nuclear receptor subfamily 3, group C, member 1 (glucocorticoid receptor)	nuclear receptor	0.523	0.148
PDE1A	5136	phosphodiesterase 1A, calmodulin-dependent	enzyme	0.524	0.082
TESSP1	360226	testis serine protease 1	peptidase	0.524	0.013
GRIN2B	2904	glutamate receptor, ionotropic, N-methyl D-aspartate 2B	ion channel	0.524	0.106
ZNF24	7572	zinc finger protein 24	transcription regulator	0.525	0.139
ARPC1A	10552	actin related protein 2/3 complex, subunit 1A, 41kDa	other	0.529	0.068
LGMN	5641	legumain	peptidase	0.531	0.081
FLJ90661	146547	protease, serine, 36	peptidase	0.531	0.044
C13ORF22	10208	ubiquitin specific peptidase like 1	other	0.535	0.022
GRIN2A	2903	glutamate receptor, ionotropic, N-methyl D-aspartate 2A	ion channel	0.536	0.077
IL19	29949	interleukin 19	cytokine	0.538	0.034
BACE1	23621	beta-site APP-cleaving enzyme 1	peptidase	0.544	0.037
HGD	3081	homogentisate 1,2-dioxygenase (homogentisate oxidase)	enzyme	0.544	0.027
GPR81	27198	G protein-coupled receptor 81	GPCR	0.549	0.087
MICA	4276	MHC class I polypeptide-related sequence A	other	0.551	0.042
USP21	27005	ubiquitin specific peptidase 21	peptidase	0.551	0.027
PPP1R13B	23368	protein phosphatase 1, regulatory (inhibitor) subunit 13B	phosphatase	0.556	0.116
CACNA1E	777	calcium channel, voltage-dependent, R type, alpha 1E subunit	ion channel	0.556	0.087
DIABLO	56616	diablo homolog (Drosophila)	other	0.558	0.002
GLRA1	2741	glycine receptor, alpha 1	ion channel	0.559	0.049
PLGL	5342	plasminogen-like B2	peptidase	0.559	0.061
PLCB3	5331	phospholipase C, beta 3 (phosphatidylinositol-specific)	enzyme	0.560	0.022
PTP4A3	11156	protein tyrosine phosphatase type IVA, member 3	phosphatase	0.561	0.018
KIF21B	23046	kinesin family member 21B	other	0.564	0.104
HAMP	57817	hepcidin antimicrobial peptide	other	0.564	0.022
PEX6	5190	peroxisomal biogenesis factor 6	enzyme	0.565	0.046
POLH	5429	polymerase (DNA directed), eta	enzyme	0.566	0.066
CREBL2	1389	cAMP responsive element binding protein-like 2	transcription regulator	0.569	0.013
BACE2	25825	beta-site APP-cleaving enzyme 2	peptidase	0.571	0.054
RAB20	55647	RAB20, member RAS oncogene family	enzyme	0.575	0.049
CRSP2	9282	mediator complex subunit 14	transcription regulator	0.578	0.084
AIP	9049	aryl hydrocarbon receptor interacting protein	transcription regulator	0.578	0.047
CBFA2T2	9139	core-binding factor, runt domain, alpha subunit 2; translocated to, 2	transcription regulator	0.583	0.085
PDE5A	8654	phosphodiesterase 5A, cGMP-specific	enzyme	0.584	0.047
PIK3AP1	118788	phosphoinositide-3-kinase adaptor protein 1	other	0.584	0.025
MKI67	4288	antigen identified by monoclonal antibody Ki-67	other	0.586	0.023
PREP	5550	prolyl endopeptidase	peptidase	0.587	0.074
ADAM21	8747	ADAM metallopeptidase domain 21	peptidase	0.587	0.022
ATP5C1	509	ATP synthase, H+ transporting, mitochondrial F1 complex, gamma polypeptide 1	transporter	0.588	0.093
RNF130	55819	ring finger protein 130	peptidase	0.589	0.066
SORCS1	114815	sortilin-related VPS10 domain containing receptor 1	transporter	0.589	0.087
HIF1A	3091	hypoxia inducible factor 1, alpha subunit (basic helixloop-helix transcription factor)	transcription regulator	0.590	0.078
KIF23	9493	kinesin family member 23	other	0.591	0.048
NR4A1	3164	nuclear receptor subfamily 4, group A, member 1	nuclear receptor	0.592	0.076
APTX	54840	aprataxin	phosphatase	0.592	0.021
ICK	22858	intestinal cell (MAK-like) kinase	kinase	0.593	0.069
TMPRSS4	56649	transmembrane protease, serine 4 Plasma	peptidase	0.593	0.024
CAPN2	824	calpain 2, (m/II) large subunit	peptidase	0.594	0.053
HTR1F	3355	5-hydroxytryptamine (serotonin) receptor 1F	GPCR	0.596	0.068
PPM1A	5494	protein phosphatase 1A (formerly 2C), magnesiumdependent, alpha isoform	phosphatase	0.597	0.040
PRKR	5610	eukaryotic translation initiation factor 2-alpha kinase 2	kinase	0.597	0.021
PAH	5053	phenylalanine hydroxylase	enzyme	0.597	0.048
PTPRE	5791	protein tyrosine phosphatase, receptor type, E Plasma	phosphatase	0.599	0.022
RNMT	8731	RNA (guanine-7-) methyltransferase	enzyme	0.599	0.024
ACPP	55	acid phosphatase, prostate	phosphatase	0.600	0.059
CNGA3	1261	cyclic nucleotide gated channel alpha 3	ion channel	0.602	0.006
HCN1	348980	hyperpolarization activated cyclic nucleotide-gated potassium channel 1	ion channel	0.603	0.020
FABP5	2171	fatty acid binding protein 5 (psoriasis-associated)	transporter	0.604	0.028
PLA2G2D	26279	phospholipase A2, group IID	enzyme	0.605	0.021
C9ORF3	84909	chromosome 9 open reading frame 3	peptidase	0.606	0.068
FABP1	2168	fatty acid binding protein 1, liver	transporter	0.606	0.014
DMTF1	9988	cyclin D binding myb-like transcription factor 1	transcription regulator	0.609	0.018
KIFC1	3833	kinesin family member C1	other	0.611	0.040
AFG3L1	172	AFG3 ATPase family gene 3-like 1 (S. cerevisiae)	peptidase	0.611	0.019
CYLD	1540	cylindromatosis (turban tumor syndrome)	transcription regulator	0.612	0.022
PAFAH1B1	5048	platelet-activating factor acetylhydrolase, isoform Ib, subunit 1 (45kDa)	enzyme	0.613	0.060
TRPV6	55503	transient receptor potential cation channel, subfamily V, member 6	ion channel	0.613	0.029
ARH	26119	low density lipoprotein receptor adaptor protein 1	transporter	0.619	0.034
CPB1	1360	carboxypeptidase B1 (tissue)	peptidase	0.620	0.047
PDE1B	5153	phosphodiesterase 1B, calmodulin-dependent	enzyme	0.620	0.025
CPO	130749	carboxypeptidase O	enzyme	0.622	0.050
UFD1L	7353	ubiquitin fusion degradation 1 like (yeast)	peptidase	0.622	0.041
CUGBP1	10658	CUG triplet repeat, RNA binding protein 1	translation regulator	0.628	0.014
PHEX	5251	phosphate regulating endopeptidase homolog, X-linked	peptidase	0.628	0.016
ILK	3611	integrin-linked kinase	kinase	0.629	0.052
APP	351	amyloid beta (A4) precursor protein	other	0.630	0.046
CLDN12	9069	claudin 12	other	0.632	0.028
HDLBP	3069	high density lipoprotein binding protein	transporter	0.635	0.005
MGC51025	353149	TBC1 domain family, member 26	other	0.636	0.029
RAD51C	5889	RAD51 homolog C (S. cerevisiae)	enzyme	0.637	0.027
APOBEC3F	200316	apolipoprotein B mRNA editing enzyme, catalytic polypeptide-like 3F	enzyme	0.638	0.022
AR	367	androgen receptor	nuclear receptor	0.639	0.043
PPGB	5476	cathepsin A	peptidase	0.639	0.013
ADAMTS6	11174	ADAM metallopeptidase with thrombospondin type 1 motif, 6	peptidase	0.640	0.029
PPP3R2	5535	protein phosphatase 3 (formerly 2B), regulatory subunit B, beta isoform	phosphatase	0.643	0.025
PPM1E	22843	protein phosphatase 1E (PP2C domain containing)	phosphatase	0.644	0.014
CD4	920	CD4 molecule	Transmembrane receptor	0.653	0.003
PPP2R1B	5519	protein phosphatase 2 (formerly 2A), regulatory subunit A, beta isoform	phosphatase	0.654	0.016
TP53RK	112858	TP53 regulating kinase	kinase	0.662	0.018
DIA1	1727	cytochrome b5 reductase 3	enzyme	0.665	0.004
DAXX	1616	death-domain associated protein	transcription regulator	0.666	0.013
TMPRSS5	80975	transmembrane protease, serine 5	peptidase	0.668	0.014
TPSD1	23430	tryptase delta 1	peptidase	0.670	0.012

a Average percent suppression of caspase activation is shown. A value of 1 indicates no change relative to control.

b Standard error of the mean is shown.

### Gene Ontology (GO) Enrichment Analysis Suggests Neuronal Glutamate Receptor Signaling and Peptidase Activity Are Involved in Mutant Htt Toxicity

**Figure 1:**
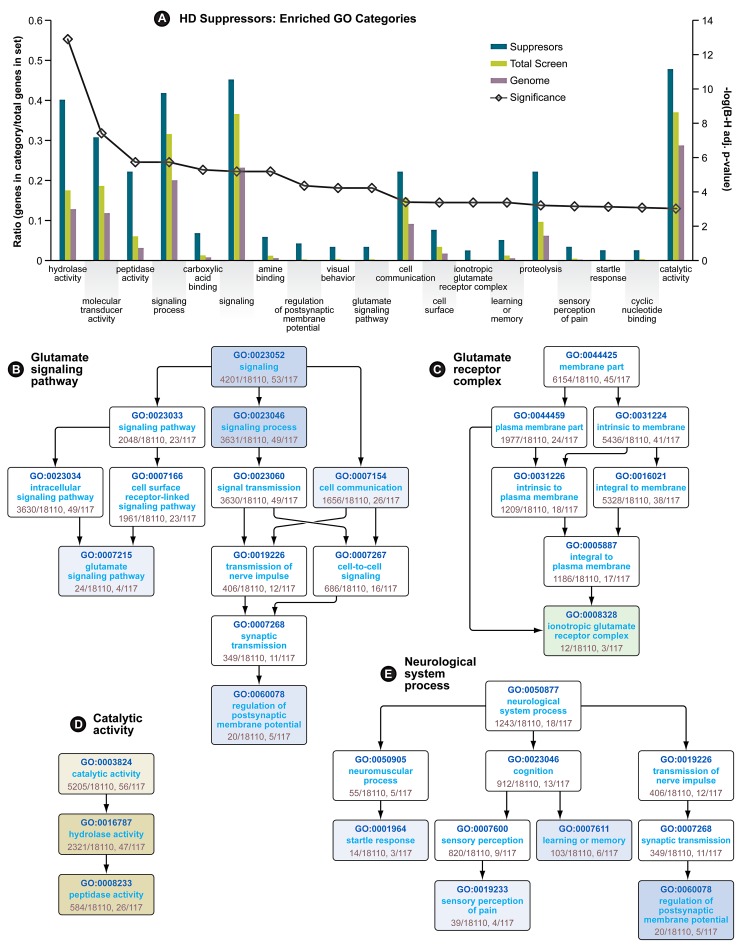
Gene Ontology Enrichment Analysis of HD Suppressors. (A) Enriched GO categories for HD suppressor genes. Significance (line with open diamonds) is represented as the −log(Benjamini-Hochberg adjusted p-value), and is scaled on the secondary axis. The remaining bars represent the ratio of genes in each category vs. genes in each dataset. (B) Directed Acyclic Graph (DAG) of Glutamate Signaling Pathway GO category. Enriched subcategories are colored blue (for Biological Process). (C) DAG of Glutamate Receptor Complex GO category. Enriched subcategories are colored green (for Cellular Component). (D) DAG of Catalytic Activity GO category. Enriched categories are colored yellow (for Molecular Function). (E) (DAG) of neurological system process GO category. In graphs B, D and E, higher significance is indicated by more intense coloration. See also Table 1.

In the primary screen performed in HEK293T cells expressing a mutant huntingtin fragment, we found that 130 siRNAs reduced caspase-3 activity to one standard deviation below the mean activity of the entire library of 7,824 (7,494 unique) siRNA pools (Table 1). Only eight siRNAs reduced caspase activity to two standard deviations below the mean. Gene ontology (GO) analysis [11] followed by a filtering step using a quality control statistic (See Materials and Methods) was used to explore the top 130 suppressors for enrichment of GO categories. Nineteen GO categories are significantly enriched among these modifiers (Figure 1A). Among these are four categories representing neurological system processes (Figure 1E), indicating that the screen for mutant Htt toxicity in the HEK293T cell model was able to identify modifiers in neuron-related pathways. Enrichment of the category “hydrolase activity” was the most significant for the suppressors (Figure 1A), and two other enriched categories, “proteolysis” and “peptidase activity” (Figure 1D) are consistent with the important role proteolytic processing plays in mutant huntingtin toxicity [12], [13]. One of the hits, siRNA targeting CASP3, directly reduces the caspase 3 toxicity readout independent of Htt. However, this hit is included in these analyses because of the direct role of CASP3 in huntingtin proteolysis [14].

We also observed glutamate signaling to be an enriched GO category among the suppressors, both as a biological process (Figure 1B), and by enrichment in members of the “ionotropic glutamate receptor complex” (Figure 1C). Evidence for the involvement of N-methyl-D-aspartate (NMDA)-type glutamate receptor excitotoxicity in HD includes heightened vulnerability of NMDA receptor-expressing neurons in HD patients [15], elevated levels of the NMDAR agonist quinolinate in the cortex and striatum of patients [16] and full-length mouse models of the disease [17] and enhanced sensitivity to NMDA in mouse models of HD [18].

### Ingenuity Pathway Analysis (IPA) Finds That the Hits Belong to Functional Categories and Canonical Pathways That Are Enriched in Neurological Disease Processes Known to Be Associated with HD

Pathway Analysis (IPA Core Analysis) was performed on the hits and enrichment of metabolic and signaling pathways, cellular and disease processes, and molecular networks were calculated (Table 2). According to the IPA analysis, the most significantly enriched functional category was “Neurological Disease”, followed by “Genetic Disorder”, both of which directly apply to HD. Additionally, siRNAs that suppressed mutant Htt toxicity were found to be enriched for genes included in the categories of “Protein Degradation”, “Nervous System Development and Function”, “Cell Death”, and “Psychological Disorders”. These all are consistent with processes known to be involved in HD. Likewise, several enriched canonical pathways, including “Amyotrophic Lateral Sclerosis Signaling”, “Synaptic Long Term Potentiation”, “Mitochondrial Dysfunction”, “Amyloid Processing”, and “Death Receptor Signaling” correlate to processes known to be involved in HD and other neurodegenerative diseases.

**Table 2:** Ingenuity Pathway Analysis (IPA) Enriched Functional Categories^a^ and Canonical Pathways for Suppressors.

Functional Category	p-value^b^	Genes
Neurological Disease	0.0000646	GRIN2A, HIF1A, PDE1A, NR3C1, APP, LGALS9, IL18R1, HRH1, AR, NR2F6, LTBR, GRIN1, GRIN2B, PAH, APTX, HRH2, PSMB5, CASP3, P2RX4, PDE9A, BACE1, HCN1, PREP, GLRA1, PDE5A, PAFAH1B1, UFD1L
Genetic Disorder	0.0000953	HAMP, GRIN2A, CD4, LHCGR, PDE1A, NR3C1, APP, LHB, PPP3R2, HRH1, AR, LDLRAP1, GRIN1, GRIN2B, PAH, APTX, HRH2, CASP3, PDE9A, BACE1, IL19, PDE5A, NAGLU, NR3C2, BACE2, CYLD, UFD1L
DNA Replication, Recombination and repair	0.0001370	APTX, PDE9A, PDE1B, PDE5A, PDE1A, NR3C1, KIFC1
Nucleic Acid Metabolism	0.0001370	PDE9A, PDEB1, PDE5A, FABP1, HIF1A, PDE1A, APP
Small Molecule Biochemistry	0.0001370	ENPEP, MAP3K14, GRIN1, GRIN2A, HAMP, GPR81, PDE9A, LHCGR, HIF1A, PDE1A, NR3C1, LHB, APP, HRH1, GAST, AR, PLA2G2D, FABP5, PDE1B, NAGLU, FABP1, PDE5A, NR3C2
Protein Degradation	0.0005570	ENPEP, GRIN2A, PSMB5, CASP3, C9ORF3, CPA1, BACE1, PHEX, PREP, LGMN, DPEP3, CAPN2, BACE2, UFD1L
Protein Synthesis	0.0005570	ENPEP, GRIN2A, PSMB5, CASP3, C9ORF3, CPA1, BACE1, HIF1A, APP, PHEX, PREP, IL19, LGMN, DPEP3, CAPN2, EIF2AK2, BACE2, UFD1L
Infectious Disease	0.0005570	PPP3R2, HRH1, GRIN1, GRIN2B, HRH2, GRIN2A, AR, CASP3, CD4, PDE5A, LTBR, NR3C1
Cancer	0.0005570	KIF23, GRIN2A, CUGBP1, CD4, LHCGR, MKI67, TP53RK, HRH1, CACNA1E, FABP1, IL1RAP, KIFC1, MAP3K14, PTPRE, HRH2, PSMB5, CASP3, HCN1, IL18BP, ATP5C1, GAST, NR3C2, CYLD, CREBL2, ENPEP, ILK, MSLN, HIF1A, LHB, NR3C1, PPP3R2, DMTF1, PLGLB2, AR, ZNF24, NR2F6, MICA, TMPRSS4, GRIN1, GRIN2B, MIPEP, C9ORF3, USP1, AIP, PPP1R3D, FABP5, NR4A1, PDE5A, EIF2AK2, PPP2R1B
Reproductive System Disease	0.0005570	GRIN2A, ILK, LHCGR, MKI67, HIF1A, LHB, NR3C1, HRH1, AR, CACNA1E, ZNF24, RAD51C, GRIN2B, GRIN1, HRH2, PTPRE, PSMB5, CASP3, C9ORF3, HCN1, ATP5C1, NR4A1, PDE5A, NR3C2, CYLD, EIF2AK2, PPP2R1B
Respiratory Disease	0.0005890	GRIN2B, GRIN1, PAH, HRH2, GRIN2A, CASP3, CD4, HIF1A, NR3C1, PPP3R2, HRH1, IL19, PDE5A, CYLD, LTBR
Gene Expression	0.0005890	MAP3K14, TPSD1, CD4, ILK, HIF1A, LGALS9, NR3C1, APP, MED14, DMTF1, GAST, AR, FABP5, NR4A1, NR3C2, LTBR, NR2F6, EIF2AK2, TNFSF13B
Nervous System Development and Function	0.0006870	GLRA2, GRIN1, GRIN2B, GRIN2A, CASP3, P2RX4, BACE1, HIF1A, APP, NR3C1, HRH1, CACNA1E, AR, GLRA1, PDE1B, PLCB3, HTR1F, NR3C2, LTBR, PAFAH1B1
Cell Cycle	0.0009920	KIF23, CD4, ILK, MKI67, HIF1A, MLL5, APP, NR3C1, DMTF1, AR, PPP1R13B, FABP1, PPM1A, LDLRAP1, RAD51C, KIFC1, TNFSF13B, PSMB5, CASP3, DAXX, GAST, NR4A1, CAPN2, EIF2AK2, CYLD, PAFAH1B1, DIABLO, POLH
Cell Death	0.0010500	CREBL2, GRIN2A, CD4, ILK, LHCGR, DLX4, HIF1A, NR3C1, LGALS9, APP, AR, PPP1R13B, CYB5R3, FABP1, PPM1A, LTBR, RAD51C, MICA, KIFC1, TNFSF13B, GRIN2B, GRIN1, PTPRE, PSMB5, CASP3, P2RX4, BACE1, TNFSF18, DAXX, IL19, PPP1R3D, GAST, PDE1B, NR4A1, EIF2AK2, CYLD, PAFAH1B1, DIABLO, PPP2R1B, POLH
Behavior	0.0014200	GRIN2B, GRIN1, UCHL3, GRIN2A, CASP3, LHCGR, HCN1, BACE1, HIF1A, APP, NR3C1, GAST, AR, GLRA1, PDE1B, NR3C2, PAFAH1B1
Cellular Development	0.0017100	CUGBP1, CASP3, CD4, PTP4A3, LHCGR, LGALS9, LHB, APP, AR, PPM1A, LDLRAP1, PAFAH1B1, RAD51C, NFSF13B
Reproductive System Development and Function	0.0017100	GRIN1, CUGBP1, AR, CASP3, LHCGR, EIF2AK2, RAD51C, PAFAH1B1, APP, NR3C1, LHB, ADAM21
Inflammatory Response	0.0021700	GRIN2A, HAMP, CD4, LHCGR, HIF1A, MLL5, NR3C1, APP, LGALS9, IL18R1, PPP3R2, HRH1, APOBEC3F, LTBR, IL1RAP, TNFSF13B, HRH2, CASP3, AIP, IL18BP, IL19, PIK3AP1, CYLD, EIF2AK2, ADAM9
Dermatological Diseases and Conditions	0.0023700	GRIN2B, GRIN1, GRIN2A, HRH2, CD4, HIF1A, APP, NR3C1, IL18BP, PPP3R2, HRH1, IL19, AR, NR4A1, PDE5A, EIF2AK2, POLH
Immunological Disease	0.0023700	GRIN2A, CD4, HGD, PDE1A, LGALS9, NR3C1, IL18R1, PPP3R2, HRH1, CPB1, AR, LTBR, IL1RAP, MICA, KIFC1, TNFSF13B, ACPP, GRIN2B, HRH2, PSMB5, MIPEP, PDE9A, C9ORF3, USP1, HDLBP, IL18BP, DAXX, IL19, ADAMTS6, GLRA1, PDE1B, NR4A1, PDE5A, PIK3AP1, EIF2AK2, DIABLO
Inflammatory Disease	0.0023700	GRIN2A, CD4, PDE1A, APP, NR3C1, IL18R1, PPP3R2, HRH1, CPB1, MICA, TNFSF13B, KIFC1, ACPP, HRH2, MIPEP, PDE9A, C9ORF3, HDLBP, USP1, IL18BP, DAXX, IL19, ADAMTS6, PDE1B, NR4A1, PDE5A, PIK3AP1, EIF2AK2
Organ Morphology	0.0031200	UCHL3, MAP3K14, AR, CASP3, LHCGR, CYLD, LTBR, LHB, NR3C1, IL1RAP, APP, TNFSF13B
Organismal Injury and Abnormalities	0.0031500	GRIN2B, GRIN1, HRH2, HAMP, GRIN2A, CD4, HIF1A, NR3C1, APP, PPP3R2, HRH1, AR, FABP1, PDE5A, HTR1F, LTBR
Psychological Disorders	0.0031900	GRIN2B, GRIN1, PAH, GRIN2A, HRH2, PDE9A, BACE1, PDE1A, APP, NR3C1, PREP, HRH1, PDE5A, UFD1L

Canonical Pathways	p-value^b^	Genes
Lysine Degradation	0.0083176	PREP, ENPEP, LGMN, PSMB5, DPEP3, CASP3, BACE1, RNF130
Amyotrophic Lateral Sclerosis Signaling	0.0104713	GRIN2B, GRIN1, GRIN2A, CACNA1E, CASP3, CAPN2
Synaptic Long Term Potentiation	0.0128825	PPP3R2, GRIN2B, GRIN1, PPP1R3D, GRIN2A, PLCB3
Mitochondrial Dysfunction	0.0288403	ATP5C1, CASP3, CYB5R3, BACE1, BACE2, APP
Lymphotoxin β Receptor Signaling	0.0288403	MAP3K14, CASP3, LTBR, DIABLO
Amyloid Processing	0.0288403	BACE1, CAPN2, BACE2, APP
Death Receptor Signaling	0.0288403	MAP3K14, DAXX, CASP3, DIABLO
Calcium-induced T Lymphocyte Apoptosis	0.0288403	PPP3R2, CD4, NR4A1, CAPN2
Induction of Apoptosis by HIV1	0.0288403	MAP3K14, DAXX, CASP3, DIABLO
cAMP-mediated Signaling	0.0331131	PPP3R2, HRH2, PDE1B, HTR1F, PDE1A, CNGA3
Circadian Rhythm Signaling	0.0346737	GRIN2B, GRIN1, GRIN2A
Cardiac β-adrenergic Signaling	0.0489779	PPP1R3D, CACNA1E, PDE1B, PDE1A, PPP2R1B

a The top 25 of 79 enriched functional categories are shown.

b Benjamini-Hochberg adjusted p-values were back calculated from −log values given by IPA by using those values as exponents to −10 (i.e. −100.05).

### IPA Network Analysis Shows Primary Interactions among HD Suppressors and Their Relationships to Htt

**Figure 2:**
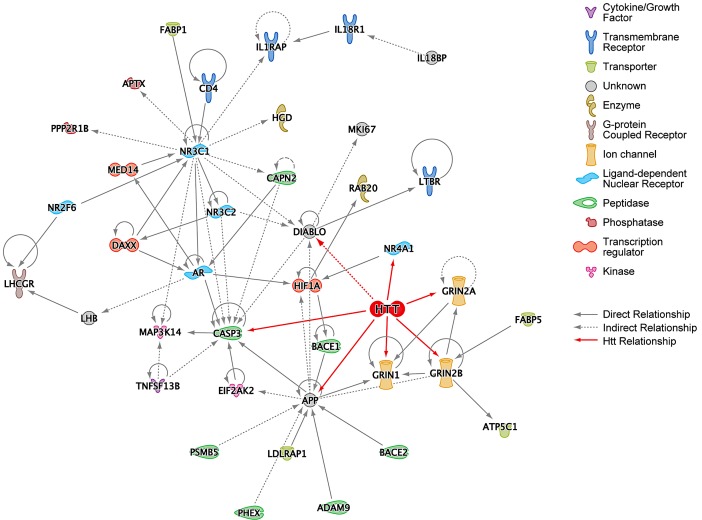
Ingenuity Pathway Analysis (IPA) of HD Suppressor Genes. (A) IPA network of the HD suppressor genes that could be directly connected to each other without intervening nodes. This network was constructed using data from all Ingenuity model organisms. Huntingtin (HTT) was manually added to this network, and its connections colored red. Functions of nodes are indicated with icons. “Direct Relationship” (solid lines) indicates direct physical contact between two molecules, *e.g.* binding or phosphorylation. “Indirect Relationship” (dotted lines) indicates a functional interaction that does not require physical contact between the two molecules, *e.g.* signaling events. See also Figure S3.

Suppressors identified in the HEK293T screen were analyzed according to several functional network criteria within IPA. First, a network was generated starting with the 130 suppressors such that a gene (node) was included in the network only if it directly connected to other genes with no intervening nodes. APP, CASP3, and NR3C1 seem to play a central role in this subnetwork of the hits, as they are the most highly connected nodes with 13, 10, and 15 connections, respectively (Figure 2). Htt was then manually added to this network, and its connections to the existing nodes introduced. All the nodes represented in Figure 2 (except for HTT) are from the 130 top hits from the siRNA screen and were included if they formed at least one connection to the subnetwork. Direct interaction of Htt to GRIN1, GRIN2A, GRIN2B, and CASP3 is intriguing because over-expression of wild-type Htt protects striatal neurons from NMDA-receptor mediated induction of caspase-3, which in turn cleaves Htt [19]. We confirmed the expression of all three of the NMDA-receptor subunits in HEK293T cells using qPCR (Figure S2).

A more inclusive network was generated using a shortest path algorithm within IPA that connects as many nodes as possible using the fewest number of intervening nodes. Restricting the analysis to data derived from “human and human tissue” results in the network shown in Figure S3. Interestingly, Htt was independently included in this network by the IPA software protocol, underscoring significant associations between the suppressors and known pathways and processes related to Htt. Similarly, a number of the shortest path connections for the toxicity suppressor hits are mediated by established components of Htt toxicity processes.

NFκB is recruited into the network as an intervening node and is known to be activated by mutant Htt through increased IkappaB kinase complex (IKK) activity [20]. IKK phosphorylates mutant Htt, enhancing its clearance [21]. Furthermore, the core kinases of IKK, IKKa and IKKbeta, have opposing roles in regulating DNA damage-induced proteolytic cleavage of Htt, with inhibition of IKKbeta blocking Htt proteolysis while increased levels of IKKa provide this benefit [22]. Detrimental activation of IKK by mutant Htt may be responsible for the increase in immune activation as indicated by elevated inflammatory cytokines such as IL6 in pre-symptomatic patients [23]. IKKbeta (IKBKB), as well as IL6, TGFB1, interferon alpha, IL2, IL15, IL18, and additional cytokines and signaling molecules were also drawn into the network as shortest path connectors. This suggests that the set of siRNA toxicity suppressors are enriched for factors involved in this inflammatory response.

Another known dysfunction in HD is transcriptional dysregulation [24]. The shortest path human network also implicated established transcription factors whose activities are affected by their physical interactions with mutant Htt. It has previously been shown that p53 (TP53) interacts with Htt *in vitro* and *in vivo* [25]. Furthermore, p53 is elevated in HD brain and in mouse models of the disease, mutant Htt upregulates p53 transcriptional activity, and inhibition of p53 prevents cytotoxicity in HD cells [26]. Similarly, Sp1 and huntingtin are known to interact [27], and mutant Htt directly represses Sp1-dependent transcription in an *in vitro* transcription system [28].

The clear identification of known modifiers of HD molecular pathology in unbiased ontology and IPA network analyses provides significant validation of the primary screening results as having relevance to established mechanisms in HD. We therefore focused on the identification and validation of novel targets and pathways not previously implicated in HD pathology.

### Multiple Components of the RRAS Signaling Pathway Modulate Mutant Htt Toxicity

**Figure 3:**
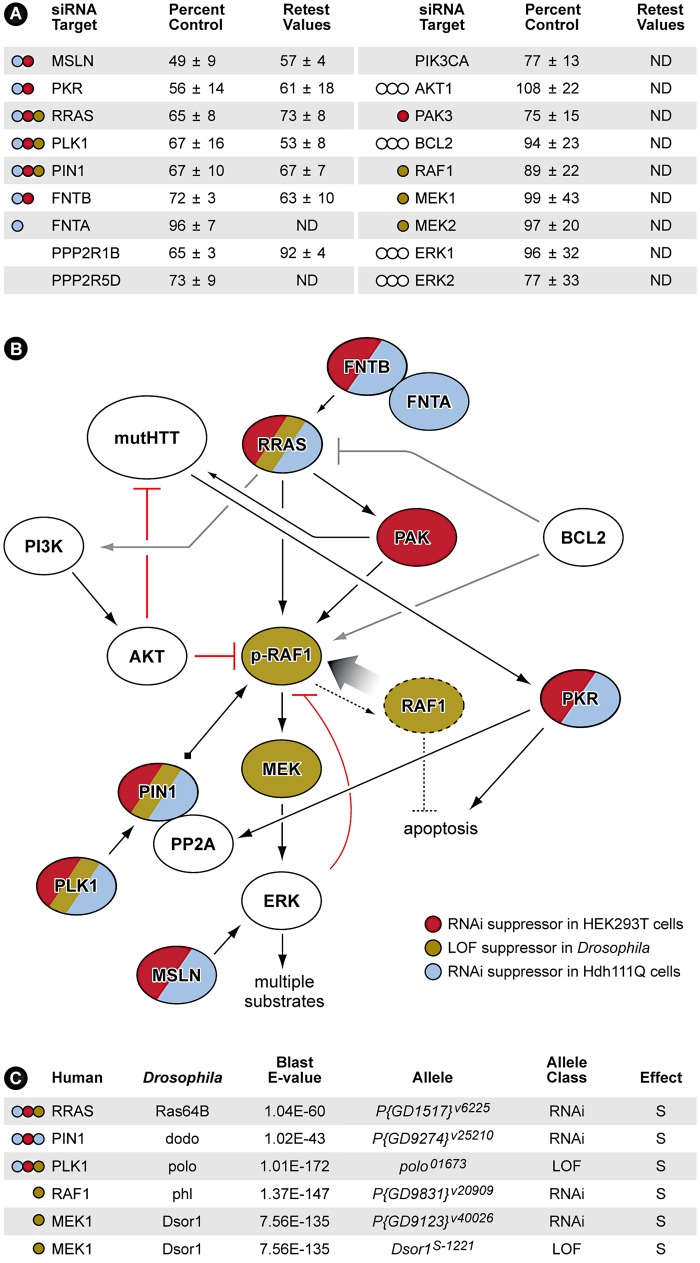
RNAi Screen Identifies Multiple Members of RRAS Signaling Cascade as Modulators of Mutant Htt Toxicity. (A) Results with siRNAs that target RRAS pathway proteins. Values are means and standard deviations observed in the primary screen and in the retest (caspase activity values are expressed as percent of non-targeting siRNA control). ND = not determined. Colored circles refer to Panel B. Additional results with relevant siRNAs are presented in Table S2. (B) Diagram showing the relationships of proteins that when inhibited in HEK293T cells (red) or ST*Hdh* ^Q111/Q111^ cells (blue) by siRNA, or in *Drosophila* by RNAi or loss-of-function (LOF) alleles (olive; see Figure 4 and Figure S5) suppress mutant Htt toxicity. Gray lines indicate relationships from published observations that may not play a role in these HD cell models. References for the pathway interconnections are presented within the text and in Text S1. (C) Modifier effects of loss-of-function in Ras pathway components on motor impairment in *Drosophila* expressing mutant Htt. S = suppressor. Colored circles refer to Panel B.

All siRNA pools conferring caspase-3 activation ≤75% of control siRNA were selected for retesting in the HEK293T assay. Those siRNAs meeting this criterion in the retest were subsequently validated in a mouse striatal-derived, full-length mutant Htt knock-in cell-based toxicity model [9]. Although not among the most stringent hits in the HEK293T cells (>1 standard deviation below the mean for the entire screen), knock-down of RRAS (related RAS viral (r-ras) oncogene homolog) [29] suppressed mutant Htt toxicity robustly, and in the more physiological mouse striatal-derived cell model, reduced toxicity to nearly the same extent as CASP3 siRNA. The retest also identified multiple components of the RRAS signaling pathway as being modifiers of mutant Htt toxicity (Figure 3A). Thus, further analyses focused on this target and the associated signaling cascade it mediates were carried out.

RRAS is a 23 kDa protein with roles in cell migration and adherence [30], apoptosis [31], neurite outgrowth [32] and hippocampal axon specification [33]. Interestingly, RRAS knockdown also suppressed the aggregation of mutant Htt in this cell-based assay (Data not shown). Mature, active proteins of the Ras superfamily are prenylated at their carboxyl-termini by farnesyltransferase or geranylgeranyl transferase enzymes. Consistent with this, we observed that siRNA inhibition of FNTB, the β subunit of the mammalian farnesyltransferase, suppressed toxicity in this screen (Figure 3A). Downstream of Ras proteins is RAF1, a MAPKKK that is phosphorylated at Serine 338 (S338) in response to activated Ras [34]. The MAPKs downstream of RAF1, ERK1/2, provide feedback inhibition by hyperphosphorylating RAF1, inactivating it [35]. To recycle RAF1 for subsequent activation, the peptidyl-prolyl cis/trans isomerase PIN1 is required for removal of inactivating phosphates by the PP2A phosphatase [35]. Polo-like kinase 1 (PLK1) stabilizes PIN1 [36] and siRNA inhibition of both PIN1 and PLK1 reduced caspase-3 induction by ∼33% relative to control (Figure 3A).

PKR (double-stranded RNA-dependent protein kinase) and MSLN (mesothelin), two additional modifiers that upon inhibition significantly suppress mutant Htt toxicity, have functions related to RRAS/RAF/MEK/ERK signaling (Figure 3B). PKR phosphorylates the B56α regulatory subunit of PP2A, increasing the phosphatase activity of the holoenzyme [37]. PKR also has a direct role in HD by binding to mutant Htt transcripts and is activated in HD brain [38]. MSLN, when overexpressed in breast cancer cells, causes sustained activation of ERK1/2 [39]. All of the RRAS signaling components identified as loss-of-function suppressors in the screen have positive roles in the signaling cascade. These data are consistent with a model indicating that pathogenically augmented signaling through RRAS contributes to mutant huntingtin-mediated toxicity (Figure 3B).

### RRAS Signaling Modulates Mutant Htt Toxicity in Mouse Cells and *Drosophila*

**Figure 4:**
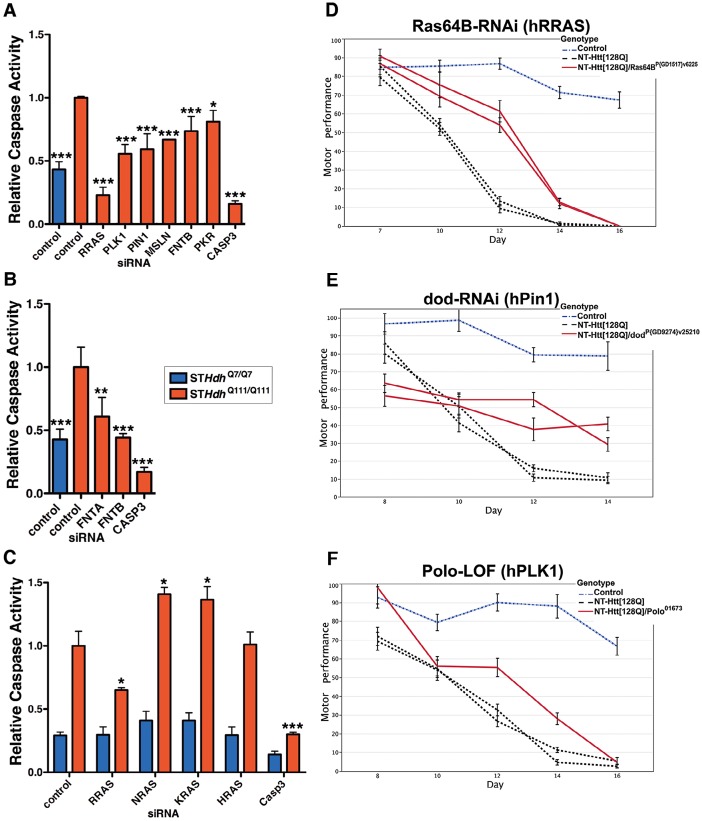
Suppression of Htt Toxicity by Knock-Down of RRAS Signaling Is Conserved across HD Models. (A) Knockdown of Ras signaling components in ST*Hdh* ^Q111/Q111^ cells (n = 3). See Figure S4A for data using individual siRNAs from deconvoluted pools. (B) siRNA targeting of subunits of the farnesyltransferase enzyme in ST*Hdh* ^Q111/Q111^ cells (n = 3). (C) Toxicity suppression is specific to RRAS knockdown among Ras family members tested (n = 3). *p<0.05, **p<0.01, ***p<0.001, ANOVA with Tukey's Multiple Comparison Test. Figure S4B shows confirmation of knockdown by western blot. (D–F) Loss-of-function in Ras signaling components Ras64B (RRAS), dod (PIN1), and polo (PLK1) suppress motor performance defects in *Drosophila melanogaster* caused by expression of mutant Htt (See Figure 3C; additional results are also presented in Figure S5). Error bars represent s.e.m.

To further validate a role for this pathway in mutant Htt toxicity in a higher content cell-based assay, we used an immortalized mouse striatal-derived cell line containing a knock-in of 111 CAGs in the mouse *Hdh* locus (ST*Hdh* ^Q111/Q111^) [9]. As in the HEK293T model, the ST*Hdh* ^Q111/Q111^ cells also exhibit enhanced caspase activation upon serum deprivation as compared to the wild-type ST*Hdh* ^Q7/Q7^ cells [40]. Knock-down of the six RRAS pathway components confirmed as suppressors in the HEK293T assay also suppressed mutant Htt toxicity in this knock-in model of HD (Figure 4A and Figure S4A). In addition, siRNAs targeting the common α subunit (FNTA) of the farnesyltransferase and geranylgeranyl transferase enzymes suppressed toxicity in ST*Hdh* ^Q111/Q111^ cells (Figure 4B). While specific targeting of RRAS is sufficient to abrogate mutant Htt-dependent toxicity, inhibition at the level of farnesylation provides similar effects, indicating that a prenylation-dependent process contributes to HD toxicity.

To determine whether the suppression effect in the ST*Hdh* ^Q111/Q111^ cells was specific to reducing RRAS levels as opposed to other Ras family members, we tested the effects of knocking down three canonical Ras proteins, NRAS, KRAS and HRAS. RRAS was the only family member tested whose knock-down suppressed toxicity in ST*Hdh* ^Q111/Q111^ cells, and suppression was specific to cells expressing mutant Htt (Figure 4C and Figure S4B). Notably, NRAS and KRAS knock-down significantly increased toxicity in the ST*Hdh* ^Q111/Q111^ cells. Knock-down of the closely related RRAS homolog RRAS2 did not result in suppression (data not shown). Modulation by RRAS in HD knock-in cells is likely independent of its antagonistic interaction with the anti-apoptotic BCL2 [31] as over-expression of BCL2 did not show an effect alone or in combination with RRAS knock-down (data not shown).

To validate these results *in vivo*, we tested RRAS signaling components in a *Drosophila* model of HD. RNAi or loss-of-function alleles for several RRAS signaling pathway components rescued the motor performance defect induced by expression of an N-terminal mutant Htt construct containing 128 glutamines in *Drosophila melanogaster* [10] (Figure 3C). Consistent with modifier effects in the two mammalian cell culture models, reduced levels of the RRAS homolog *Ras64B*, the PIN1 homolog *dodo*, and the PLK1 homolog *polo* resulted in significant rescue in *Drosophila* (Figure 4D–4F). Furthermore, decreasing the levels of either of two p21-activated kinase (PAK) proteins (*Pak* and *mbt*), which phosphorylate RAF1 in response to activated Ras [41], lead to suppression (Figure S5A–S5D). Finally, decreased amounts of *Drosophila* RAF (*polehole*), and the downstream effector MEK1 (*Dsor1*), suppressed the mutant Htt-dependent defect (Figure S5E–S5G). These results demonstrate that orthologous components of the RAS pathway modify mutant Htt-mediated motor performance defects in a whole organism model of HD. These observations further validate results obtained in the cell-based assays.

### Elevation of Activated RAF1 Observed in Cell and Mouse Models of HD

**Figure 5:**
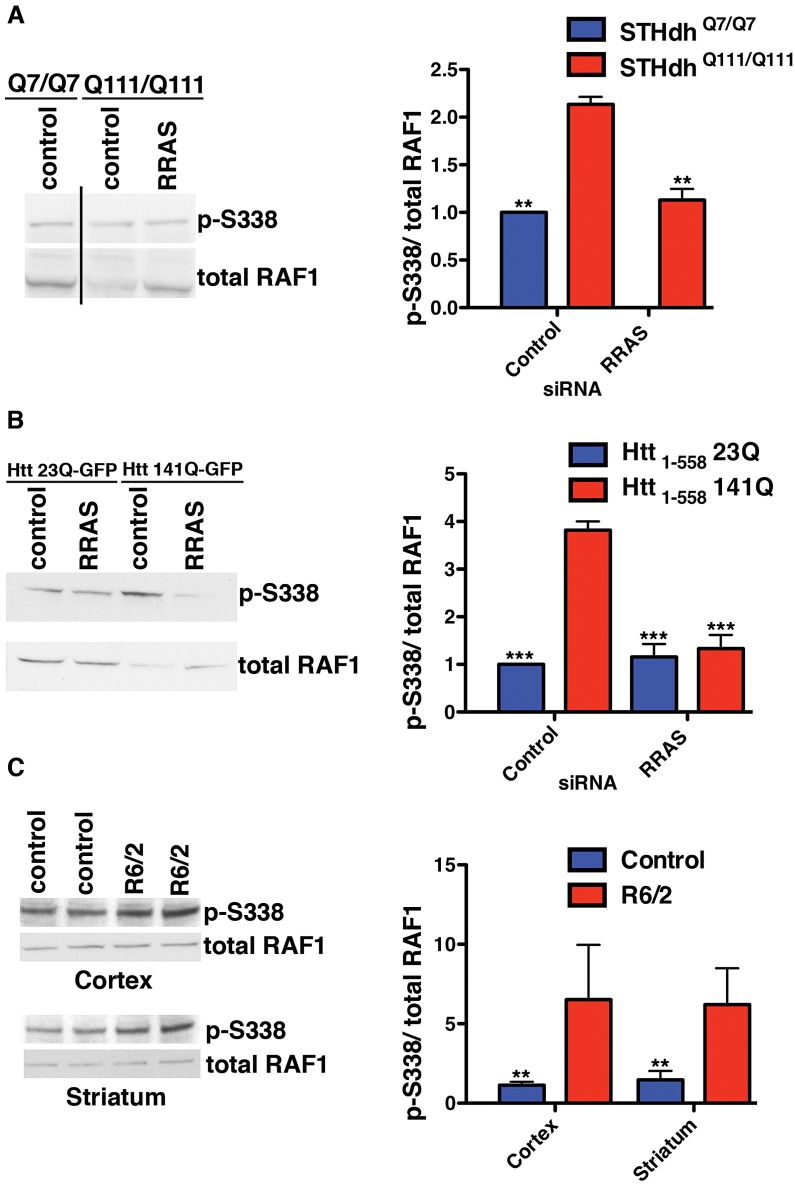
Altered RAF1 Phosphorylation in HD Models Is Rescued by RRAS Inhibition. (A) Ratio of phospho-S338 to total RAF1 is increased in ST*Hdh* ^Q111/Q111^ cells due to a reduced level of total RAF1 (n = 3). (B) Enhanced phospho-S338/total RAF1 in transiently transfected HEK293T cells (n = 3). (C) The R6/2 mouse model of Huntington's disease has elevated ratios of phospho-S338 to total RAF1 in regions of the brain affected by the disease (n = 2). **p<0.01, ***p<0.001, ANOVA with Tukey's Multiple Comparison Test (A and B), Student's ttest (C).

We investigated the mechanism by which mutant Htt interferes with normal RRAS signaling. GTP-bound RRAS binds to RAF1 [42], recruiting it to the plasma membrane and likely stimulating its activation through phosphorylation at S338 mediated by PAK proteins [41]. Activated RAF1 phosphorylates MEK1/2, which in turn activate ERK1/2 that target multiple classes of substrate [43]. Localization of RAF1 to the plasma membrane and subsequent activation of ERK phosphorylation has previously been reported to fail to protect against apoptosis [44].These toxicity suppression effects consistently resulted from reductions in proteins that are positive effectors of RRAS/RAF/MEK/ERK signaling, suggesting the pathway is pathogenically activated in HD models. This is in agreement with a previous report [45] of enhanced ERK activity in two mutant Htt cell lines, although in that study over-expression of a constitutively active mutant of MEK was protective against caspase induction. We examined the phosphorylation status of RAF1 at S338 in these cellular HD models to determine if signaling is dysregulated, as has been observed previously in Alzheimers disease (AD) patient brains [46] and a mouse model of AD [47]. The ratio of phosphorylated S338 RAF1 to total is higher in ST*Hdh* ^Q111/Q111^ cells than wild-type, and knock-down of RRAS restores the ratio (Figure 5A). In this model, the observed p-S338/total ratio in ST*Hdh* ^Q111/Q111^ cells is the result of reduced levels of unphosphorylated RAF1, as opposed to elevated p-S338 levels. Unphosphorylated RAF1 has MEK kinase-independent anti-apoptotic functions [48] (see Figure 3B), and reductions in this species could result in the release of apoptotic mediators ASK1 [49], MST2 [50] and Rok-α [51] that RAF1 normally inhibits. Hence, while activated RAF1 (as assessed by p-S338 levels) is present at comparable levels in wild-type and mutant huntingtin cells, the proportion of unphosphorylated RAF1 is decreased. RRAS inhibition restores the normal ratios of RAF1 species by increasing the level of unphosphorylated RAF1.

An effect on the p-S338/total RAF1 ratio similar to that in the knock-in HD cell model is also observed in transiently transfected HEK293T cells. Htt_1-558_141Q-GFP cells have an almost 4-fold higher ratio of p-S338 RAF1 to the total RAF1 than Htt_1-558_23Q-GFP cells (Figure 5B). RRAS siRNA abolished the difference in the ratio of p-S338/total RAF1 between cells transfected with mutant and wild-type constructs by substantially reducing the levels of p-S338 RAF1 and partially restoring the total RAF1 levels in cells with mutant Htt. RRAS knock-down in cells transfected with Htt_1-558_23Q-GFP does not alter the normal ratio, suggesting a specific effect of reducing RRAS activity in mutant Htt cells. The elevation in p-S338 RAF1 observed in this mutant Htt model, but absent in the knock-in model, may reflect greater phenotypic severity due to over-expression of a fragment of mutant Htt when compared to endogenously expressed full-length protein.

Finally, the p-S338/total RAF1 ratio was examined in the R6/2 mouse model, which expresses exon 1 of Htt with an expanded polyglutamine tract [52]. We observed elevated ratios of p-S338/total RAF1 in the striatum and cortex from R6/2 mice relative to controls, and, similar to the HEK293T fragment model, this was due to an elevation in p-S338 RAF1 (Figure 5C). The increased ratio of phosphorylated to total RAF1 species observed in the presence of mutant Htt across diverse models supports the conclusion that the RRAS signaling pathway is pathogenically modified by mutant Htt.

### Mutant Htt and RRAS Are Co-Localized in ST*Hdh* ^Q111/Q111^ Cells and in BACHD and R6/2 Mouse Models

**Figure 6:**
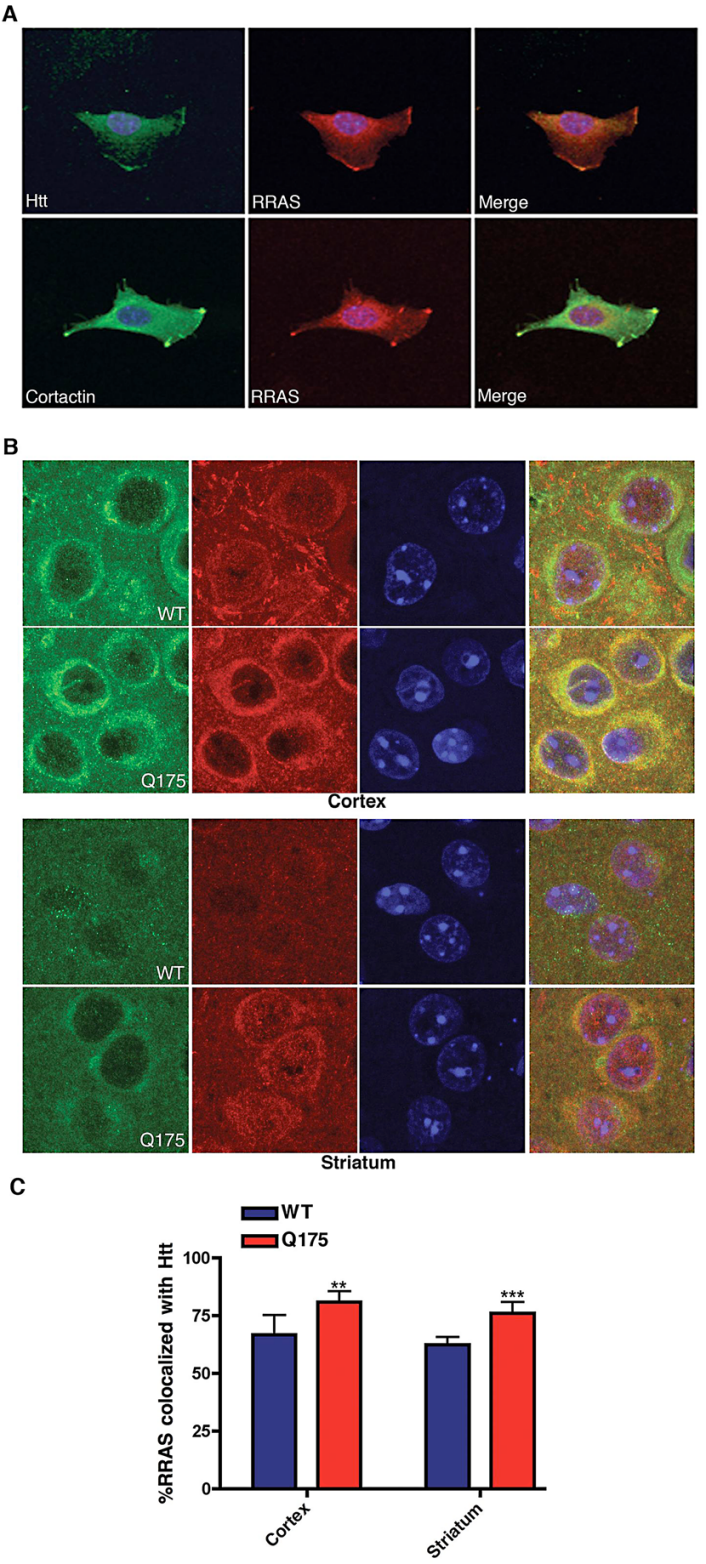
Co-Localization of huntingtin and RRas in ST*Hdh* ^Q111/Q111^ Cells and Q175 Knock-In Mouse Model. (A) Mouse ST*Hdh* ^Q111/Q111^ cell labeled with antibodies to huntingtin (upper left), RRAS (middle) and DAPI were imaged by confocal microscopy (upper panels). Lower panel shows ST*Hdh* ^Q111/Q111^ cell labeled with cortactin (lower left), RRAS (middle) and DAPI imaged by confocal microscopy. Merged images are shown (right panels). (B) Immunohistochemistry of HdhQ175 (Q175) and littermate control brain (WT) cortex and striatum stained with anti-RRAS and anti-huntingtin antibodies at 7-months of age. (C) Quantification of colocalization of RRAS with Htt. **p<0.01, ***p<.005, Student's t-test.

To examine potential mechanisms for how RRAS levels might influence mutant huntingtin toxicity we looked for co-localization of RRAS with mutant huntingtin in the mouse ST*Hdh* ^Q111/Q111^ cells. Figure 6A shows the co-localization of huntingtin and RRAS at leading edges of ST*Hdh* ^Q111/Q111^ cells (upper panels). RRAS is co-localized with the lamellipodial marker cortactin in these cells (lower panels). This localization is consistent with reported sites of RRAS localization and the known role of RRAS in cell motility and adhesion [30]. We also observed long cellular processes extending from the cell bodies in the ST*Hdh* ^Q111/Q111^ cells (data not shown). Interestingly, huntingtin protein can be seen co-localized in these regions of the cell. This suggests that the effect of RRAS levels on Htt toxicity may be due to interactions between these proteins at lamellipodia.

The immunohistochemistry of RRAS was examined in the HdhQ175 knock-in [53], R6/2 and BACHD mouse models. We found increased colocalizaton of RRAS in the striatum and cortex in all three HD models when compared to littermate controls (Figure 6B, 6C; Figure S6). We compared the distribution of RRAS in the knockin model HdhQ175 (homozygote) relative to controls since the expression level of wild-type Htt and mutant Htt are similar. We observed that the RRAS colocalized with Htt (Figure 6B, 6C) and there is a statistically significant increase in colocalization of RRAS with Htt in the HdhQ175 mouse model. We also examined the localization of RRAS in a full-length Htt mouse model with 100 CAG repeats under the control of the human Htt promoter (BACHD) using both immunohistochemistry and cellular fractionation (Figure S6). Again we found an increase in the colocalization of mutant Htt with RRAS and an increase in the membrane fraction. We conclude that the RRAS pathway is pathogenically activated by mutant Htt.

### Levels of GTP-Bound RRAS Are Elevated in ST*Hdh* ^Q111/Q111^ Cells and R6/2 Mouse Striatum

Decreased HD toxicity resulted from reductions in proteins that are positive effectors of RRAS/RAF/MEK/ERK signaling, suggesting the pathway is pathogenically activated in HD models. Direct reduction in the levels of RRAS protein using RNAi provides robust toxicity suppression in two cellular and one whole organismal model of HD. The levels of RRAS were observed to be equivalent between cells containing mutant Htt and control cells (data not shown). However, the levels of active, GTP-bound RRAS might be elevated in the presence of mutant Htt without any observable changes in the amount of total protein. GTP-bound Ras proteins bind to the Ras binding domain (RBD) of RAF1 with 100-fold or greater affinity than their GDP-bound forms [54]. This affinity increase can be used to measure the abundance of active, GTP-bound Ras proteins using pull-downs with the isolated RBD from RAF1, and then probing with antibodies against the Ras protein of interest [55].

**Figure 7:**
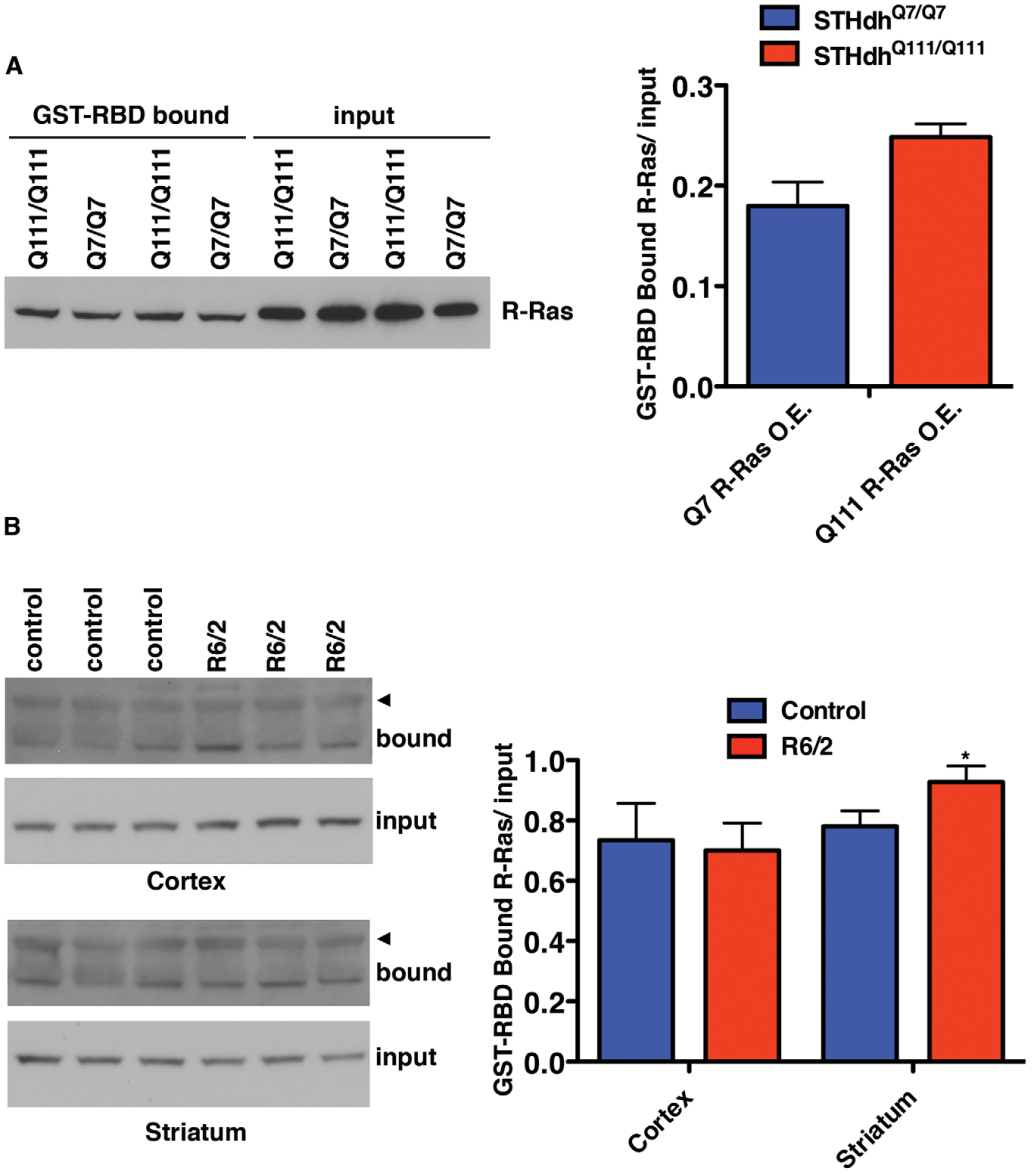
Levels of Active R-Ras Are Elevated in HD Models. (A) ST*Hdh* cells overexpressing R-Ras were subjected to GST-RBD pull-downs to detect the amount of GTP-bound R-Ras (n = 2). (B) The R6/2 mouse model of HD has increased GTP-bound R-Ras in the striatum (n = 3). The arrowheads indicate a higher molecular mass band of unknown origin that is only present in the pull-down samples. *p<0.05, Student's t-test.

Overexpression of RRAS in ST*Hdh* ^Q7/Q7^ and ST*Hdh* ^Q111/Q111^ cells was used to allow detection of GST-RBD bound RRAS. As shown in Figure 7A, ST*Hdh* ^Q111/Q111^ cells contain more active RRAS than ST*Hdh* ^Q7/Q7^ cells, although the difference is not statistically significant. This result is suggestive of an increase in the GTP-bound form of RRAS in the presence of mutant Htt.

The RBD pull-down assay was also used to measure active RRAS in the R6/2 mouse model of HD which expresses exon 1 of Htt with an expanded polyglutamine tract [52]. Homogenates of cortex and striatum from 12-week old R6/2 and control mice were subjected to GST-RBD pull-downs, followed by western blot for RRAS (Figure 7B). No significant difference in the levels of active RRAS was seen between control and R6/2 mice in the cortex samples. However, a significant (p<0.05) increase in GTP-bound RRAS is observed in R6/2 striatum relative to control, in agreement with the model of pathogenic activation of RRAS in HD.

### Small-Molecule Inhibition of Ras Signaling Suppresses Mutant Htt Toxicity

**Figure 8:**
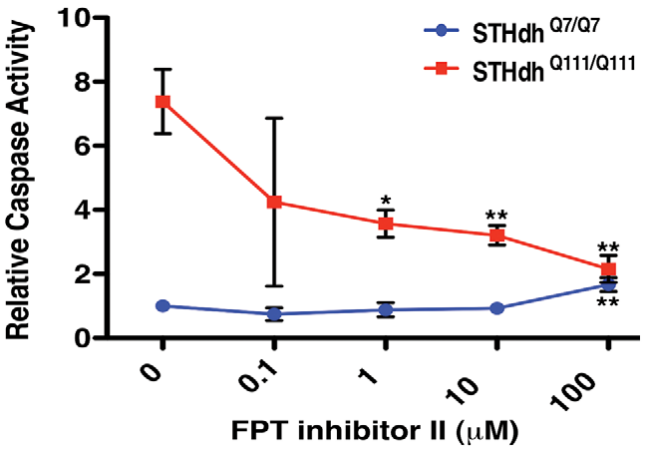
Small Molecule Inhibition of Farnesyltransferase Rescues Toxicity in an HD Cell Model. The farnesyltransferase inhibitor FPT inhibitor II rescues mutant Htt toxicity in ST*Hdh* ^Q111/Q111^ cells in a dose-dependent manner (n = 3). *p<0.05, **p<0.01, ***p<0.001, ANOVA with Tukey's Multiple Comparison Test.

Taken together, these data indicate that mutant Htt expression can pathogenically augment Ras signaling through RRAS. They suggest further that pharmacological interventions that dampen activation of Ras signaling could be of therapeutic benefit in HD. To test this idea, we examined the activity of small molecule inhibitors that target components of the Ras signaling pathway implicated by these genetic studies (see Figure 3B). Use of the *in vitro* inhibitor of RAF1 kinase activity, GW5074 in the ST*Hdh* ^Q111/Q111^ cell model reduced mutant Htt-induced toxicity (data not shown). This is in agreement with a previous report of the neuroprotective effects of this compound against a variety of toxic insults [47]. GW5074 and similar *in vitro* RAF1 kinase inhibitors function as activators of RAF1 and the related BRAF within cells [56], confounding interpretation of their toxicity rescue mechanism. Treatment with a farnesyltransferase inhibitor (FPT inhibitor II, Calbiochem) conferred robust, dose-dependent reductions in toxicity in mutant Htt cells (Figure 8). Wild-type cells were not responsive to FPT inhibitor II, supporting the hypothesis of a specific defect that is sensitive to perturbations of farnesylation in mutant Htt cells. RRAS signaling modulates toxicity in mutant Htt cells, and farnesylation of RRAS may be required for this activity. Alternative farnesyltransferase substrates such as Rhes, which has recently been shown to modulate Htt sumoylation and stability [57], may contribute to the rescue due to FPT inhibitor treatment. Farnesyltransferase inhibitors have predominantly been explored as therapeutic agents in cancer treatment, but studies have demonstrated their efficacy in a cell model of α-synuclein toxicity [58], as well as their amelioration of disease in a mouse model of progeria [59]. The beneficial effects of farnesyltransferase inhibition in these mutant Htt models suggest that pathogenic Ras signaling may be a common feature in these late onset diseases.

## Discussion

In this study we show that an unbiased siRNA screen performed in a human cell-based assay identified both known and novel targets and functional pathways as loss-of-function suppressors of mutant Htt-mediated toxicity. GO-based ontology analyses, identified glutamatergic NMDA receptors pathways as a prominent class of suppressors. Results from Ingenuity Pathway Analysis also implicated a number of other functional clusters among the hits including inflammatory processes and transcription. The dataset presented here provides a resource for interrogating the biological processes surrounding mutant Htt toxicity by indicating specific proteins and pathway components that act through these established mechanisms. Furthermore, the dataset provides significant insight into novel mechanisms and targets not previously implicated in HD pathology.

In addition to the suppressors described here, the primary screen also identified a significant number of siRNAs that enhanced caspase activity upon knock-down in the HEK293T cell-based assay. One caveat concerning RNAi enhancers of caspase activation is that modifier phenotypes may arise through general loss-of-function toxicity (e.g. knock-down of essential genes) and/or off-target effects. However, the enhancers of toxicity identified in our screen represent an interesting class of modifiers for further study.

One group of modifiers identified in the screen implicates RRAS activation as a pathogenic consequence of mutant huntingtin. This is supported by our observation that the expression of mutant huntingtin is correlated to increased levels of GTP-bound RRAS in mouse cells and in the striatum of the R6/2 HD mouse model. This is also consistent with the fact that all the suppressor effects observed in this pathway result from loss-of-function (i.e. RNAi knock-down) in positive regulators of Ras signaling. Activated RAF1 phosphorylates MEK1/2, which in turn activate ERK1/2 [43]. The alterations in RAF1 signaling observed in response to mutant Htt expression are consistent with a previous report [45] of enhanced ERK activity in two mutant Htt cell lines, although in that study over-expression of a constitutively active mutant of MEK was protective against caspase induction. Elevated RAF1 S338 phosphorylation has been reported previously in Alzheimer's disease (AD) patient brains [46] and a mouse model of AD [47]. GW5074, an *in vitro* RAF1 kinase inhibitor, has been shown to have neuroprotective effects in an AD model [47] and against a variety of toxic insults [60]. The beneficial effect of RNAi-mediated inhibition at multiple distinct points of the pathway demonstrates that loss-of-function is the mechanism responsible for rescue in HD models.

In addition to activating RAF1 and other downstream effectors such as MEK and ERK, RRAS is known to affect cell motility, and its activity is inhibited by semaphorin-plexin signaling [61]. It has recently been shown that RRAS influences integrin-dependent motility through regulation of integrin internalization in Rab11 containing vesicles [62]. Intriguingly, defects in Rab11 dependent vesicle trafficking have been implicated as a pathogenic effect of mutant huntingtin expression [63]. It will be of interest to explore how effects on semaphorin signaling and/or vesicle trafficking may play a role in the modifier effect of RRAS on mutant huntingtin toxicity. However, our modifier data from cellular, *Drosophila* and mouse models of HD indicate that aberrant signaling through RAF1 is clearly a mechanism involved in toxicity suppression by loss-of-function in RRAS. Of note the signaling defect may be distinct for RRAS in the straitum while RAF1 appears to affect the cortex and striatum. This may be a limitation in detecting RRAS activation. We show that mutant huntingtin is co-localized with RRAS in a perinuclear region as well as at the cell periphery at lamellipodia. The co-localization suggests that mutant huntingtin may exert some direct effect on RRAS through protein interaction or presence in a shared protein complex. The fact that this co-localization occurs in multiple cell compartments further suggests that mutant huntingtin could influence the function of RRAS in the context of cell migration and/or vesicle traffic as well as through signaling via RAF1.

One model for the pathogenic effect of RRAS activation is the depletion of non-phosphorylated RAF1. RAF1 has MEK kinase-independent anti-apoptotic functions [48] (see Figure 3B), and reductions in this species could result in the release of apoptotic mediators ASK1 [49], MST2 [50] and Rok-α [51] from RAF1 inhibition. Toxicity suppression resulting from inhibition of the pathway at points upstream of RAF1 phosphorylation is in agreement with this model. Additionally, RRAS and FNTB inhibition by siRNA, and pharmacologic farnesyltransferase inhibition, all reduce the amount of SDS-insoluble mutant huntingtin (data not shown). This suggests that RRAS signaling may modulate Htt solubility or turnover, and this may play a role in reduced HD toxicity. Farnesyltransferase inhibitors have demonstrated efficacy in a cell model of α-synuclein toxicity [58], as well as in amelioration of disease in a mouse model of progeria [59]. The beneficial effects of farnesyltransferase inhibition in mutant Htt models suggest that these inhibitors may have efficacy across these late onset diseases.

In this study we show that an unbiased siRNA screen performed in a human cell-based assay identified RRAS and multiple downstream signaling components as a coherent group of loss-of-function suppressors. Notably, we do not observe suppression through knock-down of other canonical Ras family members, HRAS, KRAS and NRAS, indicating a specific role for RRAS in HD. We demonstrate further that modifier effects observed in two cell-based models of HD also modulate a *Drosophila* model of HD induced motor dysfunction. In agreement with these genetic modifier effects, RRAS signaling defects associated with mutant Htt expression in cell models and the R6/2, HdhQ175 knockin and BACHD mouse model of HD are observed. Finally, we demonstrate that chemical inhibition of the Ras modifying enzyme farnesyltransferase can rescue mutant Htt toxicity in cell models of HD. The results presented here provide evidence that augmented signaling through RRAS may be a pathogenic feature of HD and that pharmacological manipulation of the Ras signaling pathway should be considered as a therapeutic strategy for the treatment of Huntington's disease.

## Materials and Methods

### Plasmids, Cell Lines, Reagents

Constructs expressing the first 558 amino acids of Htt with 141Q or 23Q and a C-terminal GFP tag (Htt_1-558_23Q-GFP or Htt_1-558_141Q-GFP) were generated by PCR amplification from pTet-splice-full-length Htt [64] using primers F: 5′-AAAGGTACCATGGAGCAGAAACTCATCTCTGAAGAG-3′ (which is upstream of the Htt coding sequence in an N-terminal myc epitope tag) and R: 5′-AAAGGATCCGACGAGGCCTGGGTCCCATCATT-3′, digested with Kpn1 and BamH1 and then sub-cloned into the pEGFP-N1 vector (Vector Biolabs). Due to significant contraction of the CAG repeats in the Htt_1-558_141Q-GFP, the vector was subsequently digested with EcoR1 and EcoRV to remove the N-terminal 530 amino acids containing the contracted CAG region and replaced with the N-terminal region from the original pTet-splice-full-length Htt148Q vector. The CAG tract length was confirmed by sequencing. HEK293T cells were used for the primary screen, and ST*Hdh^Q111/Q111^* and ST*Hdh^Q7/Q7^* (WT) cell lines [9] were used as a full-length Htt cell-based model. For the HEK293T screen, cells were plated into 96-well format using a Multidrop 384 (Thermo Electron), and all transfection and assay manipulations were carried out on a Bio Mek FX (Beckman Coulter) automated workstation.

The siRNAs used were SMARTPOOLs of the Human Druggable Genome siRNA set (Dharmacon), consisting of 76 plates of Druggable Genome siRNAs, 10 plates of Protein Kinase siRNAs, 7 plates of G-protein Coupled Receptor siRNAs, and 7 plates of Protease siRNAs. siRNAs were reconstituted in 1X siRNA buffer (Dharmacon) at a concentration of 20 µM. Mouse siRNAs were reconstituted at a concentration of 1 µg/µl.

### HEK293T and ST*Hdh* ^Q111/Q111^ Transfections

For the initial screen toxicity suppression assays, transfections were performed by plating 20,000 HEK293T cells in 100 µl media into each well of a collagen I coated 96-well plate (BD Biosciences) and, 24 h after plating, adding a mixture of 25 µl serum-free media, 320 ng DNA, 17 pmoles siRNA, and 1 µg Lipofectamine 2000 (Invitrogen). Screening of the Druggable Genome set of 76 plates was performed with 10 siRNA plates on a given day, transfecting each plate of siRNAs plates of cells in triplicate. The Kinase, GPCR and Protease sets were done similarly, except the Protease set was screened in quadruplicate. In the initial ST*Hdh* ^Q111/Q111^ assay (Figure 4A), nucleofection was performed with a Nucleofector 96-well Shuttle System (Amaxa Biosystems) using program FF-130 and Solution SG with 200,000 cells/well and 40 picomoles of siRNA. All other mouse cell nucleofections were carried out with 3 µg siRNA and two million ST*Hdh^Q111/Q111^* or ST*Hdh^Q7/Q7^* cells using a Nucleofector II device with Solution L and program T-030, plating 50,000 cells per well in a 96 well plate. For both cell types, the media was removed and replaced with 100 ul of serum-free media 48 h after transfection. At 72 h after transfection, the cells were assayed for caspase 3/7 activity.

### Caspase 3/7 Assays

For the HEK293T siRNA screen, media was removed by inverting 96-well plates and replacing with 100 µl (50 µl in the case of the Protease set) of a 50/50 mixture of serum-free DMEM and APO 3 HTS 1X Lysis Buffer (Cell Technology, Inc). Plates were shaken for 30 s at 700 rpm. Plates were incubated at room temperature for 20 minutes to ensure complete lysis, and then centrifuged at 2,500× g for 3 min. Three 10 µl aliquots were taken to assay for protein (BCA assay; Pierce). To the remaining 70 µl (20 µl for the Protease set) of lysate, 30 µl of a DMEM/1x lysis buffer mixture containing DTT [15 mM]_final_ and substrate (zDEVD_2_Rhodamine 520) were added. Plates were again shaken for 30 s, and then assayed for fluorescence (EX_485 nm_/Em_530 nm_) in a Fusion alpha HTS plate reader (PerkinElmer) with reads every 44 min. Assays were the same for ST*Hdh* cells except that cells were lysed in 50 µl and reads were taken every 51 s. All assays were performed in triplicate (quadruplicate for Protease siRNA screen) for each transfected siRNA, and caspase 3/7 activity was calculated as the change in RFU per minute per milligram of total protein in the well.

### Criterion for Mutant Htt Toxicity Suppressor “Hit” Classification

Each plate in the primary HEK293T screen contained three of each of the following control wells: co-transfection of Htt_1-558_23Q-GFP with non-targeting (NT) siRNA or CASP3 siRNA (toxicity controls), Htt_1-558_141Q-GFP with NT siRNA (negative control), or Htt_1-558_141Q-GFP with CASP3 siRNA (positive control). Caspase 3 activity units were first normalized for position effects by dividing the values for a given well by the average for that well throughout the plates screened on that day (28–30 plates). These were then represented as fractions of the “negative control” caspase activity values derived from control wells present on the individual plate. This normalization was chosen instead of plate mean because the arrangement of plates in the siRNA sets is by gene families, and thus not organized with an unbiased distribution. The mean and standard error for each siRNA were then calculated for the normalized individual replicates. siRNAs were selected as “hits” if the confidence interval, defined as the mean value for each siRNA ± its standard error, was less than one standard deviation (0.317) from the mean (1) of all siRNAs in the entire screen. siRNAs were thus considered hits if the sum of their normalized caspase 3 activity mean and standard error was less than 0.683 (1−0.317).

#### Q–PCR

Total RNA was extracted from HEK293T cells using the RNeasy Kit (Qiagen) as per the manufacturer's instructions. 200 ng of total RNA was reverse-transcribed using the Reverse Transcription PCR kit (Applied Biosystems). Q-PCR was performed using SYBR Green (Applied Biosystems) on the Light Cycler 490 system. For quantification the threshold cycle Cp of each amplification was determined by the 2nd derivative analysis provided by the LightCycler 480 software and the 2−ΔΔCp method was used to determine the relative expression level of each gene normalized against the house-keeping gene β-actin. The specificity of each pair of primers was tested by comparing to a negative control sample of water on both quantification analysis and high-resolution melting curve analysis. PCR products were then run on an 2% agarose gel to confirm product size. The primers used are: GRIN1 F: 5′-CCTACAAGCGGCACA-AGG-3′, R: 5′-TCAGTGGGATGGTACTGCTG-3′; GRIN2A F: 5′-TTGCTTCAGTTT-GTGGGTGA -3′, R: 5′-GGTGTGGCAGATCCCAGTG-3′; GRIN2B F: 5′-AGCAATGGGACTGTCTCACC-3′, R: 5′-AACATCATCACCCATACG TCA G-3′.

### Gene Ontology Enrichment Analysis

The genes encoding the 130 hits were analyzed for enrichment in gene ontology (GO) categories in order to gain insight into the biological context of mutant Htt toxicity suppression. The enrichment analysis was performed using Ontologizer [11], a java-based program that performs enrichment analysis in a manner that takes into account parent-child relationships in the GO tree, rather than the standard term-for-term analysis that does not incorporate this feature of GO. For this analysis, the genome was used as the background (population) dataset, and the hits as the test (study) set. Due to a lack of power, the screened siRNA library could not be used as the background dataset. The Parent-Child Union algorithm in Ontologizer was used for analysis and the data was represented as Benjamini-Hochberg (B-H) corrected p-values. A threshold of *p*≤0.05 was applied to the GO enrichment results and Ontologizer was used to generate directed acyclic graphs (DAGs) of the enriched GO terms. To ensure that the enriched categories were truly enriched over the siRNA library, the same analysis was repeated using the siRNA library list as the study set. The enriched categories for the toxicity suppression hits were then compared against the same categories in the siRNA library. From this information, proportions for each analysis were generated for each GO category: number of genes in the GO category/number of genes in the study set. These proportions were then subjected to a two-tailed proportions test as follows:
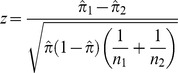
where
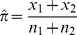
and where 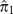 and 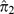 are the sample proportions, 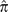 is the combined proportion, *x* is the number of hits within a population, and *n* is the total number of samples in the population. The resulting z-scores were compared against the z-distribution and p-values calculated. Categories that did not pass a significance threshold of *p*≤0.05 were excluded from the results. Additionally, only GO categories below level 2 were included, and any categories referring to non-eukaryotic processes were excluded.

### Ingenuity Pathway Analysis

In order to further classify the biological context of the mutant Htt toxicity suppressor hits, the dataset was analyzed using Ingenuity Pathway Analysis (IPA; Ingenuity® Systems, www.ingenuity.com). The dataset was subjected to an IPA Core Analysis using all model organisms and the Ingenuity Knowledge base as the background. Functional categories and canonical pathways were then represented as B-H adjusted p-values and were considered to be enriched if *p*≤0.05.

A network analysis of the mutant Htt toxicity suppressor hits was also performed within IPA. For these analyses, three approaches were utilized. First, a network was created from the hits that could be directly connected to each other using orthologous data from all model organisms within IPA. Subsequently, the Huntingtin (HTT) protein was manually added to this pathway in order to observe its potential connections. The second network was created by linking together as many hits as possible using the “shortest path” mechanism from within IPA, in which intervening nodes are placed in the network to connect the hits to each other. Generation of this second network was limited to human data. The third network was generated in the same fashion as the second, but incorporating all model organisms as the background.

#### *Drosophila* motor performance assay

The Elav-GAL4; UAS:128QHtt[F33A] fly has been described previously [10]. Two populations of 15 adult virgin females per genotype were scored for their ability to climb 9 cm in 15 s. Ten trials for each population were performed at each timepoint and the percentage of flies that climbed for each day was plotted. Flies were raised at 26.5°C, transferred to vials containing new food every day, and tested at the same time of day to exclude circadium rhythm effects. Control animals (Elav-GAL4 driver alone) were compared to flies expressing nervous system-restricted mutant Htt (NT-Htt[128Q]) and to NT-Htt[128Q] flies crossed to lines with reduced levels of Ras pathway components. Strains were obtained from the Bloomington *Drosophila* Stock Center of the Vienna *Drosophila* RNAi Center.

#### Western blot determination of p-S338/total RAF1 ratios

For western blot analysis, HEK293T cells were co-transfected with the indicated siRNA and Htt_1-558_-GFP constructs using Lipofectamine 2000 with the ratios described above, but with proportionate amounts onto cells plated into 6-well plates. For ST*Hdh* cells, nucleofection was carried out as described above and 600,000 cells were then plated into a well of a 6-well plate. HEK293T cells were lysed in M-PER (Pierce) containing Complete Protease Inhibitor Cocktail, EDTA-free and PhosSTOP Phosphatase Inhibitor Cocktail (both from Roche) after 48 h, and ST*Hdh* cells after 24 h, as these timepoints showed maximal knockdown of RRAS in the cell line under study. Following a 10 min, 14,000× g centrifugation, the protein concentration of the supernatant was measured using the BCA method (Pierce), and 15 µg of protein was loaded into each lane of a NuPAGE 4–12% Bis-Tris Gel (Invitrogen). The gels were transferred to 0.2 µm nitrocellulose, and the membranes then probed with 1∶500 of either anti-phospho-Raf-1 (Ser338) (Upstate, #05-538) to detect p-S338 RAF1, or anti-Raf1 (clone Y198, abcam, #ab32025) to detect total RAF1. Following secondary HRP-conjugated antibody incubation and chemiluminescence reagent addition, membranes were exposed to film. Membranes were then stripped and re-probed with a second antibody to determine the levels of the other species of RAF1. Densitometry was performed using a GS-710 Densitometer (BioRad) and Quantity One Software.

For R6/2 and control brain analyses, striatum and cortex were extracted from euthanized animals and dounce homogenized and sonicated in 300 µl (striatum) or 200 µl (cortex) T-PER (Pierce) with protease and phosphatase inhibitors. Following BCA protein determination, 40 µg were loaded per lane and processed as above.

#### Immunocytochemistry of Hdh^111Q/111Q^ cells

Cells were fixed in 4% PFA in PBS for 20 min, washed for 10 min in 1X PBS and 10 min in 1X TBS. Cells were permeabilized in 0.25% triton in 1X TBS for 15 minutes and washed twice in 1X TBS for 5 min. Cells were blocked for 1 hr at room-temp with 10% normal goat serum in TBS with chicken anti-ms IgG at 1∶500. Incubation with the primary antibodies, MAB2166 (Millipore) and RRAS (Abcam) was performed overnight at 4 degrees in 1% BSA in TBS. Cells were then washed 3 times for 10 min in 1X TBS and incubated with secondary antibodies in 1% BSA for 1 hr at room temp. MAB2166 (Millipore 1∶200) was labeled with Alexa 555 nm donkey anti-mouse (Molecular Probes) and RRAS antibody (1∶250, Abcam) was labeled with Alexa 488 nm donkey anti-rabbit. Cells were washed 3×5 min in 1X TBS and mounted in Prolong Gold (Invitrogen) with DAPI. The cells were imaged on a Zeiss LSM 510 NLO.

#### Immunohistochemistry of R6/2

Paraffin-embedded tissue was de-paraffinized (2×5 min xylene), re-hydrated (2×100%, 2×95%, 80%, 70% EtOH, 4 min each) and then rinsed in PBS, pH 7.3 for 10 min. Antigen retrieval was performed in 10 mM citrate buffer (pH 6.0) by microwaving on high for 2 min and then 20% power for an additional 5 min. Slides were left to cool 15 min on benchtop and then rinsed 10 min in PBS. Block buffer (2% normal goat serum, 0.1% BSA, 0.3% TX-100 in PBS) w/1∶500 chicken anti-mouse IgG was applied for 30 min, RT and then primary antibodies (1∶250 RRAS. abcam, ab47536 and 1∶200 Htt, Chemicon, MAB2166) in block buffer were left on sections ON at 4°C. After 3×10 min PBS washes, sections were incubated in secondary antibodies (Alexa) for 1 hr at RT: 1∶500 goat anti-mouse 555 nm and 1∶500 goat anti-rabbit 488 nm antibody (RRAS/Htt MAB2166). After 3×10 min PBS washes, sections were cover-slipped in Prolong Gold with DAPI (Molecular Probes).

#### RRAS/Htt immunofluorescence in BACHD

WT and BACHD mice were paraffin-embedded and sectioned. They were deparaffinized with xylene and rehydrated through a series of ethanol dilutions. Antigen retrieval was performed in 10 mM citrate buffer by microwaving for 2 minutes at maximum power and then 20% power for another 5 minutes, letting cool on the bench top for 20 minutes. Chicken anti-mouse IgG (2 µg/ml, Aves Labs, Tigard, OR) was added to the block buffer and incubated for 30 minutes at room temperature. Slides were incubated with polyclonal RRAS (1∶50, ab47536 abcam) and Htt 2166 (1∶100, Millipore) ON at 4°C. Next day, slides were washed for 2 hours and incubated in secondary antibodies Alexa Flour 488 goat anti-rabbit IgG and Alexa Flour 555 goat anti-mouse IgG (1∶500, Invitrogen) for 1 hour at RT followed by another 2 hour wash and coverslipping with Prolong Gold containing DAPI (Invitrogen). Colocalization of RRAS to Htt was quantified using Imaris X64 7.3.0 software.

#### RRAS/Htt immunofluorescence in HdhQ175 homozygote knock-in

WT and Q175 homozygote mice were paraffin-embedded and sectioned. They were deparaffinized with xylene and rehydrated through a series of ethanol dilutions. Antigen retrieval was performed in 10 mM citrate buffer by microwaving for 2 minutes at maximum power and then 20% power for another 5 minutes, letting cool on the bench top for 20 minutes. Chicken anti-mouse IgG (2 µg/ml, Aves Labs, Tigard, OR) was added to the block buffer and incubated for 30 minutes at room temperature. Slides were incubated with monoclonal RRAS (1∶50, AT3728a abgent) and Anti-Huntingtin [3–16 aa] (1∶50, H7540 Sigma) ON at 4°C. Next day, slides were washed for 2 hours and incubated in secondary antibodies Alexa Flour 488 goat anti-rabbit IgG and Alexa Flour 555 goat anti-mouse IgG (1∶500, Invitrogen) for 1 hour at RT followed by another 2 hour wash and coverslipping with Prolong Gold containing DAPI (Invitrogen). Colocalization of RRAS to Htt was quantified using Imaris X64 7.3.0 software.

### Fractionation of Striatum

Striatum was dissected from Q175 homozygote and BACHD mice along with WT littermates at 7-months and 9-months respectively. The mice striatum were homogenized in fractionation buffer (50 mM HEPES pH 7.0, 200 mM NaCl, 5 mM MgCl_2_, 1 mM DTT, 1 mM EDTA, 0.25M Sucrose containing protease inhibitors) by using a glass dounce for 60 strokes twice with 1-minute rest in between. Samples were then centrifuged at 2,000×g for 10 min at 4°C. The nuclear pellet (P1) was saved and the post nuclear supernatant (S1) was further centrifuged at 100,000×g for 1 hr at 4°C in a Beckman-Coulter TLA-100 rotor. The supernatant (S2, cytosol) was saved and the total membrane pellet (P2) was re-suspended in fractionation buffer supplemented with 1% Triton X-100 and went through a freeze thaw cycle at −80°C.

#### Active RRAS pull-down from ST*Hdh* cells overexpressing RRAS and mouse striatum and cortex

ST*Hdh* cells were nucleofected with 2 ug of RRAS overexpression plasmid using the conditions described above for siRNA nucleofection. The Active Ras Pull-Down and Detection Kit (Cat# 89855; ThermoScientific) was used on equivalent amounts of protein from cell lysates according to the manufacturer's instructions except that anti-RRAS antibody (Cat# ab47536; Abcam) was used to detect the pulled-down RRAS.

Striatum and cortex from 12-week old R6/2 and age-matched control mice were dounce homogenized (60 strokes, 2×) in T-PER (Pierce) supplemented with Complete Protease Inhibitor Cocktail, EDTA-free and PhosSTOP Phosphatase Inhibitor Cocktail (both from Roche) and 5 mM MgCl_2_. Homogenates were then sonicated at 40W 5 times for 5 seconds each pulse, and cleared by a 15 minute and then 5 minute 16,000×g centrifugation at 4°C. Supernatants were assayed for protein concentration by BCA assay (Pierce), and 1 mg of soluble protein from each sample was used for the Active Ras Pull-Down and Detection Kit according to the manufacturer's instructions except that anti-RRAS antibody was used to detect the pulled-down R-Ras.

#### Compound treatment of ST*Hdh* ^Q111/Q111^ and ST*Hdh* ^Q7/Q7^ cells

Cells were plated at 25,000 cells per well into 96-well collagen-coated plates. After 24 h, FPT Inhibitor II (Calbiochem) or vehicle (water) was added to cells for an additional 24 h of pre-treatment, followed by a final 24 h incubation in serum-free media containing the same concentration of FPT Inhibitor II or water. Caspase 3/7 activity was measured as above.

## Supporting Information

Figure S1 Toxicity of Mutant Htt-GFP Construct. Toxicity controls for primary screen in HEK293T cells transiently transfected with Htt_1-558_141Q-GFP. The y-axis is the caspase 3/7 activity (in change in RFU per minute per milligram of total protein). The columns are the means of the four controls (Htt_1-558_23Q-GFP with control siRNA or CASP3 siRNA, and Htt_1-558_141Q-GFP with control siRNA or CASP3 siRNA) over the eight batches of screens. Error bars are standard deviation. ***p<0.001, n = 8, ANOVA with Tukey's Multiple Comparison Test.
(TIF)

Figure S2 HEK293T cells express GRIN1, GRIN2A and GRIN2B. Total RNA from HEK293T cells was harvested and analyzed for GRIN expression using Q-PCR. Expression was normalized to actin controls and products were run on an agarose gel to confirm product size.
(TIF)

Figure S3 Shortest Path Network of All Mutant Htt Toxicity Suppressors. IPA Network of the HD suppressor genes connected to each other using the shortest path possible between the majority of the genes in the HD suppressors list. Red connections are NMDA receptor (GRIN) related interactions, purple are APP related interactions, blue are CASP3 related interactions, and green are NFkB related interactions. Genes that were included in the HD suppressors gene list are shaded gray, while all others are white.
(EPS)

Figure S4 Controls for siRNA Knock-down Experiments. (A) Deconvolution of Dharmacon siRNA SMARTPOOLS targeting the indicated Ras signaling components in ST*Hdh* ^Q111/Q111^ cells. Western blots with the indicated antibodies show the degree of knockdown of the targeted protein with the indicated siRNA duplex. “control” is an siRNA duplex that targets luciferase. Caspase 3/7 activity measurements of the nucleofected cells from part a demonstrating that at least two duplexes from each pool provide significant toxicity suppression. The enhanced toxicity of PIN1 #4 is likely an off-target effect as knock-down of PIN1 by the other three duplexes from the pool results in toxicity suppression. (B) Knock-down of Ras family proteins in ST*Hdh* ^Q111/Q111^ and ST*Hdh* ^Q7/Q7^ cells using Dharmacon SMARTPOOLS. The indicated siRNA treated samples were evaluated for knockdown with the indicated antibodies. We did not detect an appropriately migrating band for K-Ras in the ST*Hdh* ^Q111/Q111^ cells, but the results with the ST*Hdh* ^Q7/Q7^ cells confirm that the siRNA targets the appropriate gene product.
(TIF)

Figure S5 *Drosophila* Strains that Suppress a Mutant Htt-Dependent Motor Performance Deficit. (A–C) Motor performance assays for the indicated mutant lines tested over different days are shown (see Figure 3C). Details are presented in Materials and Methods.
(TIF)

Figure S6 Co-localization of huntingtin in R6/2 and BACHD mouse brain. (A) Immunohistochemistry of R6/2 and littermate control brain stained with anti RRAS and anti-huntingtin antibodies at 12-weeks of age. (B) Immunohistochemistry of BACHD and littermate control (NTg) cortex and striatum stained with anti RRAS and anti-huntingtin antibodies at 12-months of age. (C) Quantification of colocalization of RRAS with Htt. (D) Western blot of cellular fractionation (total membrane fraction P2) of BACHD and littermate control (NTg) striatum using RRAS antibody at 9-month of age (upper panel). Quantification of RRAS in membrane fraction (P2). (E) Fractions total lysate (input), P1, S1, P2 and S2. Western blot was probed with RRAS.
(PDF)

Table S1 Complete Results for HEK293T Screen. Complete results for primary siRNA caspase activation screen are presented. Average caspase 3/7 activation is indicated as a percent of non-targeting control siRNA (1.0 indicates no change).
(XLS)

Table S2 Results with Additional Isoforms and Components of R-Ras/RAF/MEK/ERK Pathway. Effects of siRNA knock-down of select R-Ras/RAF/MEK/ERK pathway components on caspase activation in ST*Hdh* ^Q111/Q111^ cells are shown.
(DOCX)

Text S1 Published Supporting Evidence for Figure 3B and Supporting Materials and Methods.
(DOCX)
