# Supplementary figures and images for: A Genome-Scale RNA–Interference Screen Identifies RRAS Signaling as a Pathologic Feature of Huntington's Disease
Source: PLoS Genet. 2012 Nov 29;8(11):e1003042. doi: 10.1371/journal.pgen.1003042 (PMC3510027; doi:10.1371/journal.pgen.1003042)

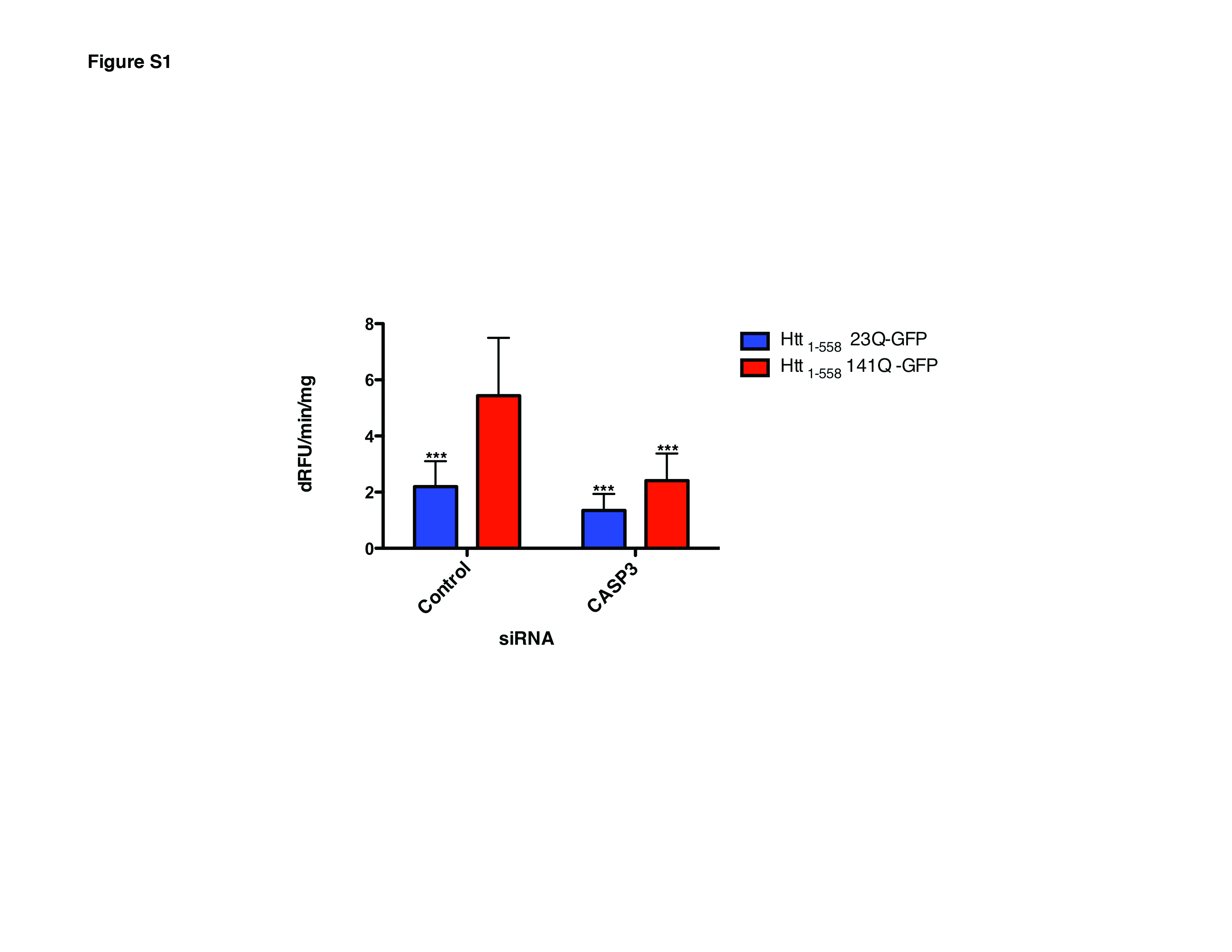

Supplement: Figure S1 — Toxicity of Mutant Htt-GFP Construct. Toxicity controls for primary screen in HEK293T cells transiently transfected with Htt1-558141Q-GFP. The y-axis is the caspase 3/7 activity (in change in RFU per minute per milligram of total protein). The columns are the means of the four controls (Htt1-55823Q-GFP with control siRNA or CASP3 siRNA, and Htt1-558141Q-GFP with control siRNA or CASP3 siRNA) over the eight batches of screens. Error bars are standard deviation. ***p<0.001, n = 8, ANOVA with Tukey's Multiple Comparison Test. (TIF) [file pgen.1003042.s001.tif]

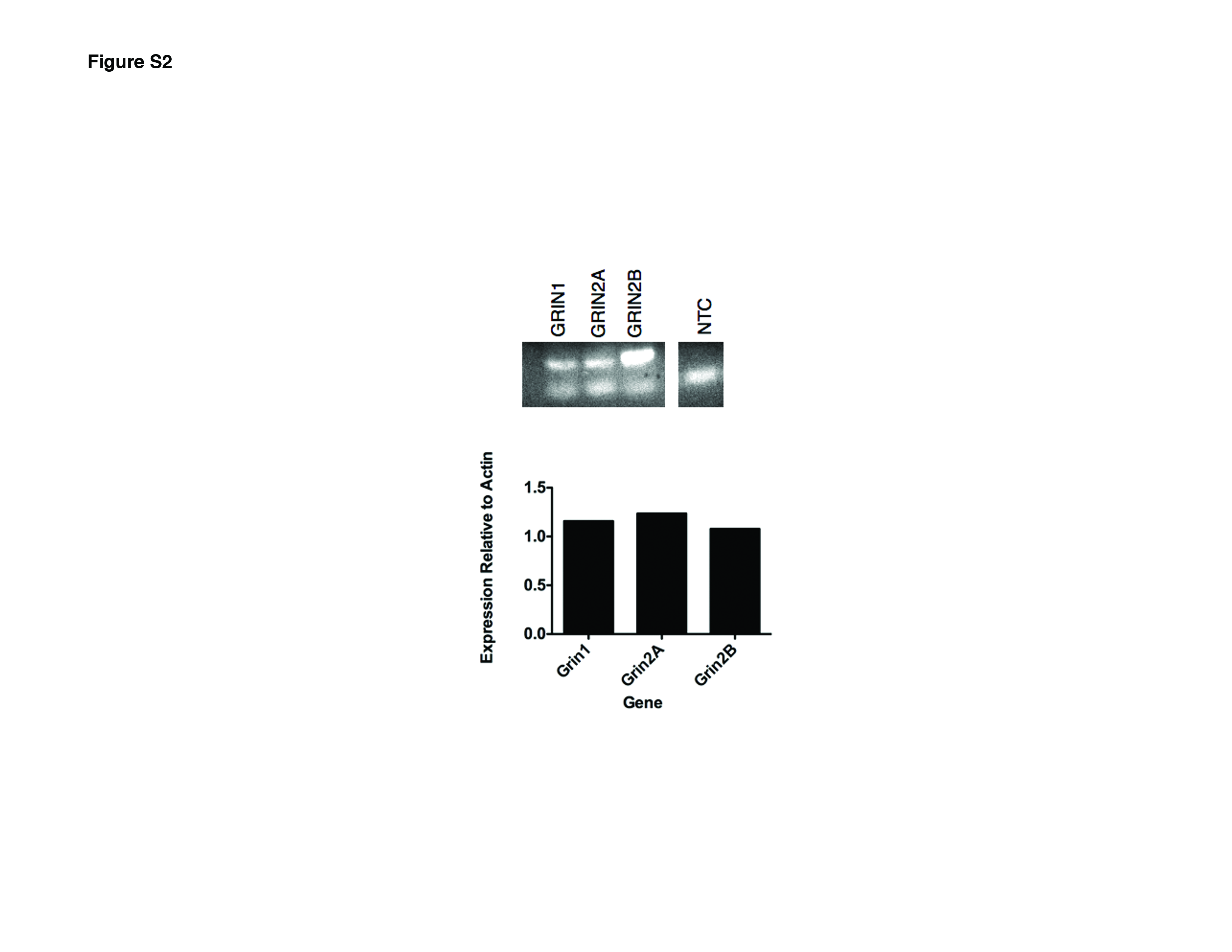

Supplement: Figure S2 — HEK293T cells express GRIN1, GRIN2A and GRIN2B. Total RNA from HEK293T cells was harvested and analyzed for GRIN expression using Q-PCR. Expression was normalized to actin controls and products were run on an agarose gel to confirm product size. (TIF) [file pgen.1003042.s002.tif]

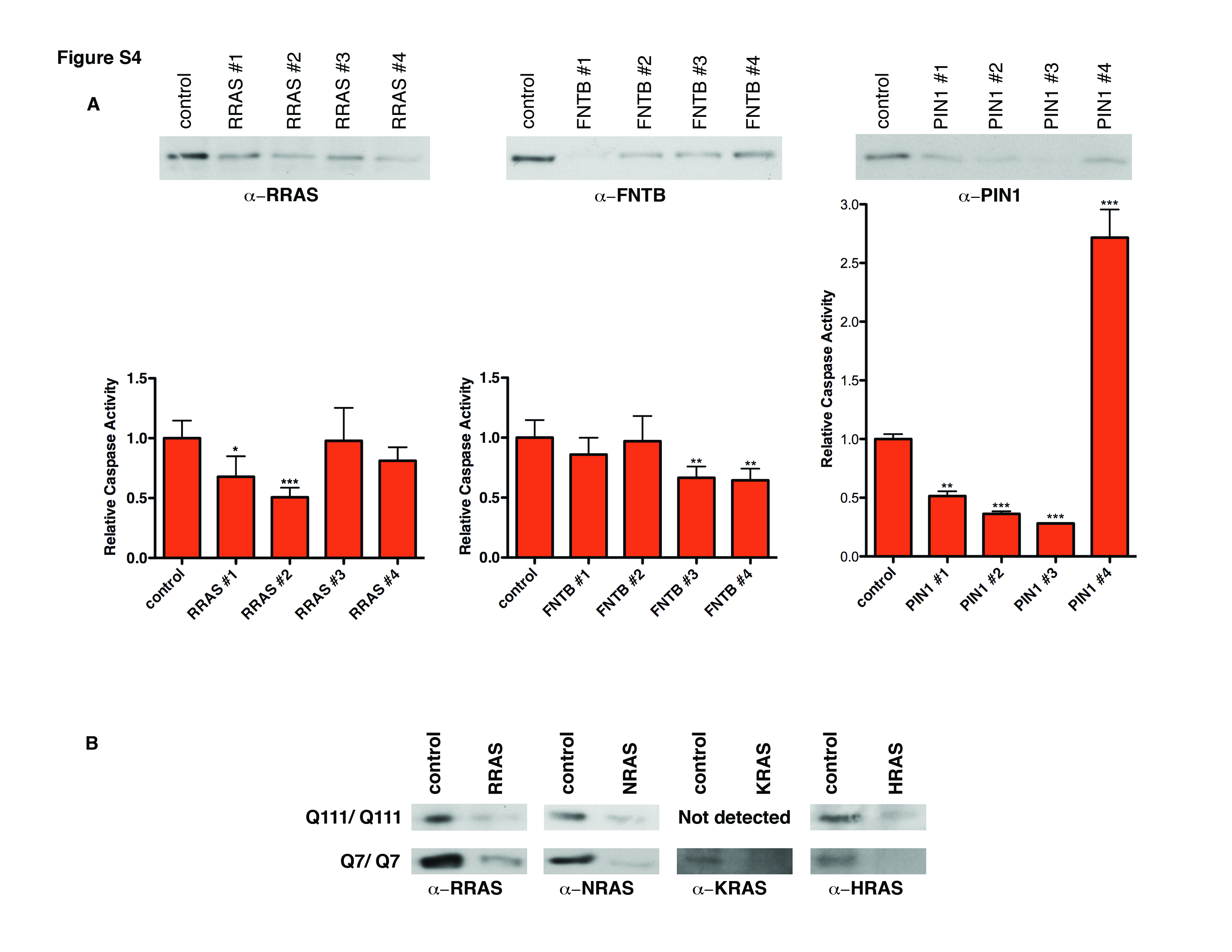

Supplement: Figure S4 — Controls for siRNA Knock-down Experiments. (A) Deconvolution of Dharmacon siRNA SMARTPOOLS targeting the indicated Ras signaling components in STHdh Q111/Q111 cells. Western blots with the indicated antibodies show the degree of knockdown of the targeted protein with the indicated siRNA duplex. “control” is an siRNA duplex that targets luciferase. Caspase 3/7 activity measurements of the nucleofected cells from part a demonstrating that at least two duplexes from each pool provide significant toxicity suppression. The enhanced toxicity of PIN1 #4 is likely an off-target effect as knock-down of PIN1 by the other three duplexes from the pool results in toxicity suppression. (B) Knock-down of Ras family proteins in STHdh Q111/Q111 and STHdh Q7/Q7 cells using Dharmacon SMARTPOOLS. The indicated siRNA treated samples were evaluated for knockdown with the indicated antibodies. We did not detect an appropriately migrating band for K-Ras in the STHdh Q111/Q111 cells, but the results with the STHdh Q7/Q7 cells confirm that the siRNA targets the appropriate gene product. (TIF) [file pgen.1003042.s004.tif]

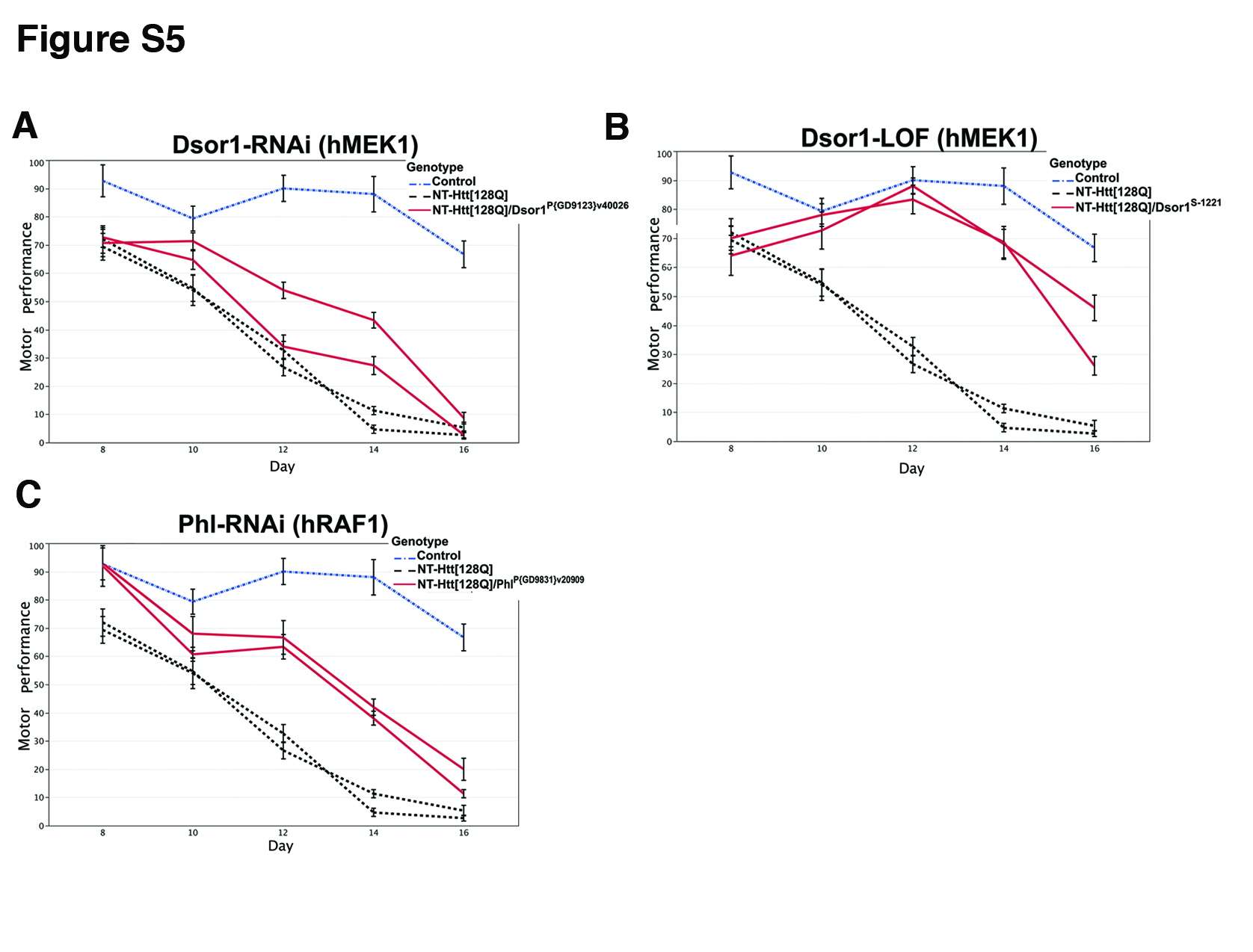

Supplement: Figure S5 — Drosophila Strains that Suppress a Mutant Htt-Dependent Motor Performance Deficit. (A–C) Motor performance assays for the indicated mutant lines tested over different days are shown (see Figure 3C). Details are presented in Materials and Methods. (TIF) [file pgen.1003042.s005.tif]

Supplemental Figure 6

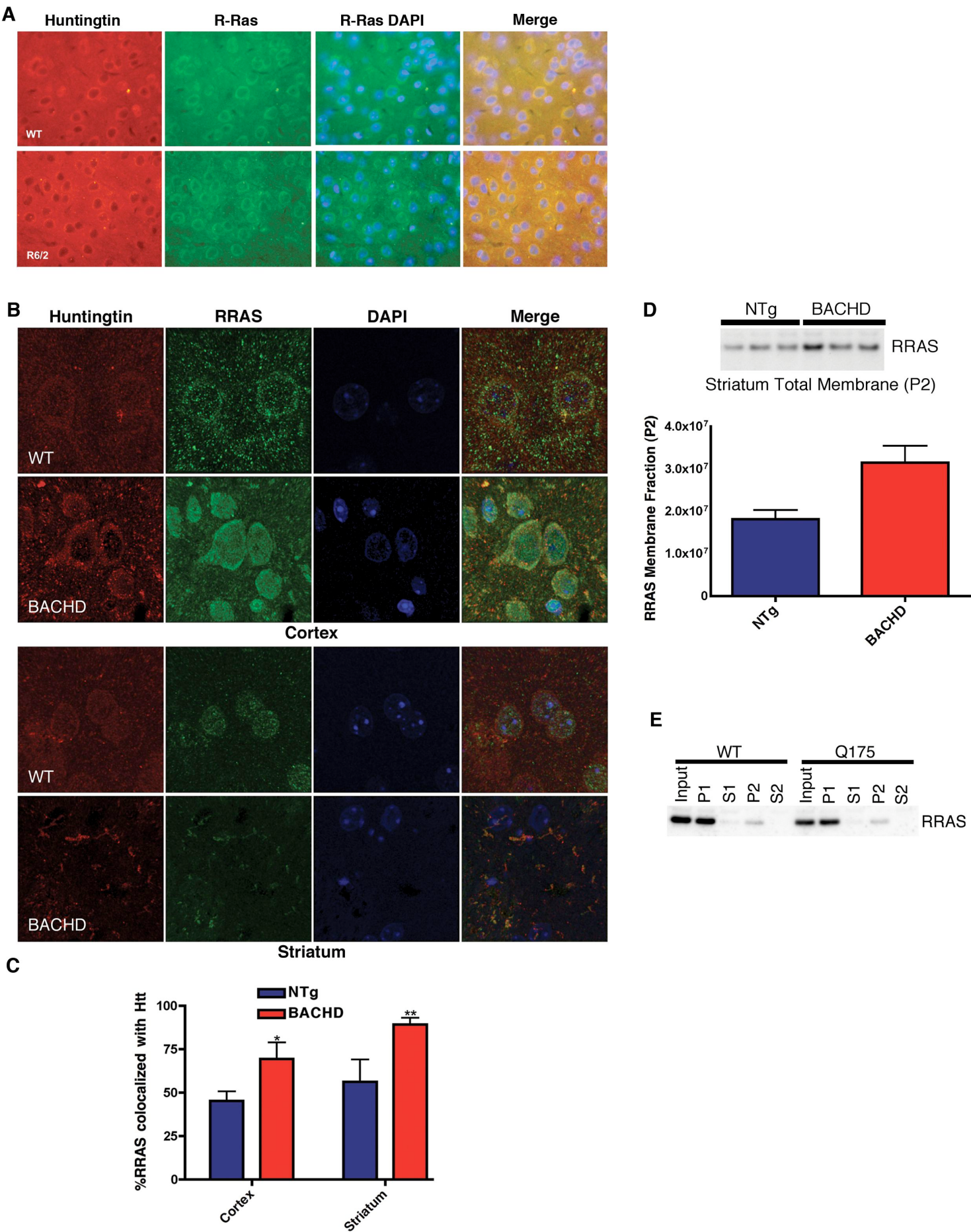

Supplement: Figure S6 — Co-localization of huntingtin in R6/2 and BACHD mouse brain. (A) Immunohistochemistry of R6/2 and littermate control brain stained with anti RRAS and anti-huntingtin antibodies at 12-weeks of age. (B) Immunohistochemistry of BACHD and littermate control (NTg) cortex and striatum stained with anti RRAS and anti-huntingtin antibodies at 12-months of age. (C) Quantification of colocalization of RRAS with Htt. (D) Western blot of cellular fractionation (total membrane fraction P2) of BACHD and littermate control (NTg) striatum using RRAS antibody at 9-month of age (upper panel). Quantification of RRAS in membrane fraction (P2). (E) Fractions total lysate (input), P1, S1, P2 and S2. Western blot was probed with RRAS. (PDF) [file pgen.1003042.s006.pdf]
