# Supplementary material for: A Genome-Scale RNA–Interference Screen Identifies RRAS Signaling as a Pathologic Feature of Huntington's Disease
Source: PLoS Genet. 2012 Nov 29;8(11):e1003042. doi: 10.1371/journal.pgen.1003042 (PMC3510027; doi:10.1371/journal.pgen.1003042)
Supplement: Table S2 — Results with Additional Isoforms and Components of R-Ras/RAF/MEK/ERK Pathway. Effects of siRNA knock-down of select R-Ras/RAF/MEK/ERK pathway components on caspase activation in STHdh Q111/Q111 cells are shown. (DOCX) [file pgen.1003042.s008.docx]

**Table S2. Results with Additional Isoforms and Components of R-Ras/ RAF/ MEK/ ERK Pathway.** Values are mean ± standard deviation, n = 3.

| **siRNA TARGET** | **PERCENT CONTROL** | **siRNA TARGET** | **PERCENT CONTROL** |
| --- | --- | --- | --- |
| AKT2 | 119 ± 41 | PPP2R1A | 76 ± 3 |
| AKT3 | 115 ± 42 | PPP2R2A | 102 ± 12 |
| PGGT1B | 97 ± 31 | PPP2R5B | 90 ± 13 |
| PIK3C2A | 110 ± 23 | PPP2R5C | 101 ± 10 |
| PIK3C2B | 83 ± 21 | PPP2R5E | 86 ± 3 |
| PIK3C2G | 93 ± 11 | HRAS | 89 ± 9 |
| PIK3C3 | 129 ± 10 | KRAS2 | 110 ± 28 |
| PIK3CB | 80 ± 18 | NRAS | 83 ± 24 |
| PIK3CD | 105 ± 18 | ARAF1 | 123 ± 19 |
| PIK3CG | 135 ± 45 | BRAF | 85 ± 22 |
| PIK3R1 | 114 ± 56 | ASK1 | 92 ± 37 |
| PIK3R2 | 95 ± 20 | ROCK1 | 110 ± 34 |
| PIK3R3 | 108 ± 26 | ROCK2 | 82 ± 15 |
| PIK3R4 | 96 ± 40 | STK3 | 119 ± 49 |
| PPP2CA | 81 ± 21 | STK4 | 109 ± 70 |
| PPP2CB | 87 ± 13 |  |  |
