# Supplementary material for: A Genome-Scale RNA–Interference Screen Identifies RRAS Signaling as a Pathologic Feature of Huntington's Disease
Source: PLoS Genet. 2012 Nov 29;8(11):e1003042. doi: 10.1371/journal.pgen.1003042 (PMC3510027; doi:10.1371/journal.pgen.1003042)
Supplement: Text S1 — Published Supporting Evidence for Figure 3B and Supporting Materials and Methods. (DOCX) [file pgen.1003042.s009.docx]

**Text S1**

**Published supporting evidence for Figure 3B.** Huntington’s disease tissues show increased PKR activity [1]. PKR positively regulates PP2A [2]. A dominant-negative PKR mutant inhibited apoptosis induced by ASK1 [3]. R-Ras is reported to activate phosphoinositide 3-kinase (PI3K) in COS-7 and NIH/3T3 cells [4], but we did not observe an involvement of this activation in the cell models used in the current study (data not shown). PI3K activates Akt [5,6], and Akt has been shown to be neuroprotective against the toxic insult of mutant Htt [7]. In addition, Akt can negatively regulate RAF1 in regards to its MEK kinase activity [8,9]. R-Ras contains a prenylation motif (CAAX sequence) that has been shown to be important for its membrane localization and function in integrin activation [10]. The R-Ras CAAX motif, C-V-L-L, by virtue of the fact that its X position is leucine, is predicted to be a substrate for geranylgeranylation. However, farnesylation of this sequence by the heterodimeric farnesyltransferase enzyme [11] is also possible [12-14]. R-Ras interacts with BCL2 [15] and has been reported to promote apoptosis in growth factor-deprived cells through a BCL2-suppressible mechanism [16]. However, we did not observe BCL2-dependent toxicity suppression in the HD knock-in cell model (data not shown). When GTP-bound, R-Ras directly interacts with RAF1 [17], but activating phosphorylation of RAF1 is mediated by Src and p21-associated (Cdc42/Rac) kinase (PAK) proteins [18]. Among the PAK family of proteins, PAK1 [19], PAK3 [20], and PAK7/PAK5 [21] have been shown to phosphorylate RAF1. PAK1 is also reported to interact with soluble mutant Htt and promote its toxicity [22], while wild-type Htt is reported to interact with PAK2 and inhibit its apoptosis-promoting cleavage [23], a function that could be defective in the mutant protein. PAK1 phosphorylation has been shown to direct RAF1 to mitochondria where it protects cells from the intrinsic pathway of apoptosis [18]. Mitochondrial localization of RAF1, in contrast to plasma membrane localization, results in protection from apoptosis, and BCL2 mediates RAF1 localization to mitochondria [24]. This BCL2-based mechanism may not be involved in our study because BCL2 overexpression did not suppress toxicity (data not shown). However, there are distinct MEK kinase-independent functions of RAF1 that inhibit apoptosis [25-28] that may be involved. Activation by phosphorylation of RAF1 results in phosphorylation of MEK [29,30], in turn activating its kinase activity towards ERK [31]. ERK engages in feedback inhibition by phosphorylating RAF1 at five or more sites, inactivating its ability to respond to further Ras-mediated stimulation [32]. PIN1 and PP2A cooperate to remove these inhibitory phosphorylation modifications, returning RAF1 to an activation-competent state [32]. PLK1 stabilizes PIN1 by phosphorylating it and inhibiting its ubiquitination and subsequent degradation [33]. Finally, overexpression experiments suggest MSLN promotes sustained activation of ERK [34].

**Supporting Materials and Methods**

**siRNA pool** **deconvolution experiments.** Nucleofection of individual siRNA duplexes was performed as described in Materials and Methods except that 750 ng (R-Ras and FNTB) or 500 ng (PIN1) of the individual siRNA was used, and compared to the same amount of a luciferase-targeting control siRNA duplex (Dharmacon, cat. # D-001210-02). Western blots were as described in Methods. Antibodies used were anti-R-Ras (abcam ab47536), anti-FNTB (Upstate, Cat# 09-121), and anti-PIN1 (abcam, Cat# ab54505).

**Ras family knockdown experiments.** Western blots were performed as described in Methods. Antibodies used were anti-R-Ras (abcam ab47536), anti-N-Ras (Santa Cruz Biotechnology, Cat# sc-31), anti-K-Ras (Santa Cruz Biotechnology, Cat# sc-30), and anti-H-Ras (ab32417).

**Supporting Information References**

1. Peel AL, Rao RV, Cottrell BA, Hayden MR, Ellerby LM, et al. (2001) Double-stranded RNA-dependent protein kinase, PKR, binds preferentially to Huntington's disease (HD) transcripts and is activated in HD tissue. Hum Mol Genet 10: 1531-1538.

2. Xu Z, Williams BR (2000) The B56alpha regulatory subunit of protein phosphatase 2A is a target for regulation by double-stranded RNA-dependent protein kinase PKR. Mol Cell Biol 20: 5285-5299.

3. Takizawa T, Tatematsu C, Nakanishi Y (2002) Double-stranded RNA-activated protein kinase interacts with apoptosis signal-regulating kinase 1. Implications for apoptosis signaling pathways. Eur J Biochem 269: 6126-6132.

4. Marte BM, Rodriguez-Viciana P, Wennstrom S, Warne PH, Downward J (1997) R-Ras can activate the phosphoinositide 3-kinase but not the MAP kinase arm of the Ras effector pathways. Curr Biol 7: 63-70.

5. Burgering BM, Coffer PJ (1995) Protein kinase B (c-Akt) in phosphatidylinositol-3-OH kinase signal transduction. Nature 376: 599-602.

6. Franke TF, Yang SI, Chan TO, Datta K, Kazlauskas A, et al. (1995) The protein kinase encoded by the Akt proto-oncogene is a target of the PDGF-activated phosphatidylinositol 3-kinase. Cell 81: 727-736.

7. Humbert S, Bryson EA, Cordelieres FP, Connors NC, Datta SR, et al. (2002) The IGF-1/Akt pathway is neuroprotective in Huntington's disease and involves Huntingtin phosphorylation by Akt. Dev Cell 2: 831-837.

8. Rommel C, Clarke BA, Zimmermann S, Nunez L, Rossman R, et al. (1999) Differentiation stage-specific inhibition of the Raf-MEK-ERK pathway by Akt. Science 286: 1738-1741.

9. Zimmermann S, Moelling K (1999) Phosphorylation and regulation of Raf by Akt (protein kinase B). Science 286: 1741-1744.

10. Oertli B, Han J, Marte BM, Sethi T, Downward J, et al. (2000) The effector loop and prenylation site of R-Ras are involved in the regulation of integrin function. Oncogene 19: 4961-4969.

11. Reiss Y, Goldstein JL, Seabra MC, Casey PJ, Brown MS (1990) Inhibition of purified p21ras farnesyl:protein transferase by Cys-AAX tetrapeptides. Cell 62: 81-88.

12. Kho Y, Kim SC, Jiang C, Barma D, Kwon SW, et al. (2004) A tagging-via-substrate technology for detection and proteomics of farnesylated proteins. Proc Natl Acad Sci U S A 101: 12479-12484.

13. Trueblood CE, Ohya Y, Rine J (1993) Genetic evidence for in vivo cross-specificity of the CaaX-box protein prenyltransferases farnesyltransferase and geranylgeranyltransferase-I in Saccharomyces cerevisiae. Mol Cell Biol 13: 4260-4275.

14. Yokoyama K, Goodwin GW, Ghomashchi F, Glomset JA, Gelb MH (1991) A protein geranylgeranyltransferase from bovine brain: implications for protein prenylation specificity. Proc Natl Acad Sci U S A 88: 5302-5306.

15. Fernandez-Sarabia MJ, Bischoff JR (1993) Bcl-2 associates with the ras-related protein R-ras p23. Nature 366: 274-275.

16. Wang HG, Millan JA, Cox AD, Der CJ, Rapp UR, et al. (1995) R-Ras promotes apoptosis caused by growth factor deprivation via a Bcl-2 suppressible mechanism. J Cell Biol 129: 1103-1114.

17. Spaargaren M, Martin GA, McCormick F, Fernandez-Sarabia MJ, Bischoff JR (1994) The Ras-related protein R-ras interacts directly with Raf-1 in a GTP-dependent manner. Biochem J 300 (Pt 2): 303-307.

18. Alavi A, Hood JD, Frausto R, Stupack DG, Cheresh DA (2003) Role of Raf in vascular protection from distinct apoptotic stimuli. Science 301: 94-96.

19. Zang M, Hayne C, Luo Z (2002) Interaction between active Pak1 and Raf-1 is necessary for phosphorylation and activation of Raf-1. J Biol Chem 277: 4395-4405.

20. King AJ, Sun H, Diaz B, Barnard D, Miao W, et al. (1998) The protein kinase Pak3 positively regulates Raf-1 activity through phosphorylation of serine 338. Nature 396: 180-183.

21. Wu X, Carr HS, Dan I, Ruvolo PP, Frost JA (2008) p21 activated kinase 5 activates Raf-1 and targets it to mitochondria. J Cell Biochem 105: 167-175.

22. Luo S, Mizuta H, Rubinsztein DC (2008) p21-activated kinase 1 promotes soluble mutant huntingtin self-interaction and enhances toxicity. Hum Mol Genet 17: 895-905.

23. Luo S, Rubinsztein DC (2009) Huntingtin promotes cell survival by preventing Pak2 cleavage. J Cell Sci 122: 875-885.

24. Wang HG, Rapp UR, Reed JC (1996) Bcl-2 targets the protein kinase Raf-1 to mitochondria. Cell 87: 629-638.

25. Chen J, Fujii K, Zhang L, Roberts T, Fu H (2001) Raf-1 promotes cell survival by antagonizing apoptosis signal-regulating kinase 1 through a MEK-ERK independent mechanism. Proc Natl Acad Sci U S A 98: 7783-7788.

26. Huser M, Luckett J, Chiloeches A, Mercer K, Iwobi M, et al. (2001) MEK kinase activity is not necessary for Raf-1 function. Embo J 20: 1940-1951.

27. O'Neill E, Rushworth L, Baccarini M, Kolch W (2004) Role of the kinase MST2 in suppression of apoptosis by the proto-oncogene product Raf-1. Science 306: 2267-2270.

28. Piazzolla D, Meissl K, Kucerova L, Rubiolo C, Baccarini M (2005) Raf-1 sets the threshold of Fas sensitivity by modulating Rok-alpha signaling. J Cell Biol 171: 1013-1022.

29. Dent P, Haser W, Haystead TA, Vincent LA, Roberts TM, et al. (1992) Activation of mitogen-activated protein kinase kinase by v-Raf in NIH 3T3 cells and in vitro. Science 257: 1404-1407.

30. Kyriakis JM, App H, Zhang XF, Banerjee P, Brautigan DL, et al. (1992) Raf-1 activates MAP kinase-kinase. Nature 358: 417-421.

31. Ray LB, Sturgill TW (1988) Insulin-stimulated microtubule-associated protein kinase is phosphorylated on tyrosine and threonine in vivo. Proc Natl Acad Sci U S A 85: 3753-3757.

32. Dougherty MK, Muller J, Ritt DA, Zhou M, Zhou XZ, et al. (2005) Regulation of Raf-1 by direct feedback phosphorylation. Mol Cell 17: 215-224.

33. Eckerdt F, Yuan J, Saxena K, Martin B, Kappel S, et al. (2005) Polo-like kinase 1-mediated phosphorylation stabilizes Pin1 by inhibiting its ubiquitination in human cells. J Biol Chem 280: 36575-36583.

34. Uehara N, Matsuoka Y, Tsubura A (2008) Mesothelin promotes anchorage-independent growth and prevents anoikis via extracellular signal-regulated kinase signaling pathway in human breast cancer cells. Mol Cancer Res 6: 186-193.
